# Photodissociation Spectroscopy and Photofragment Imaging of the Mg^+^(Benzene) Complex

**DOI:** 10.1021/acs.jpca.4c05703

**Published:** 2024-11-25

**Authors:** Jason E. Colley, Nathan J. Dynak, John R. C. Blais, Michael A. Duncan

**Affiliations:** Department of Chemistry, University of Georgia, Athens, Georgia 30602, United States

## Abstract

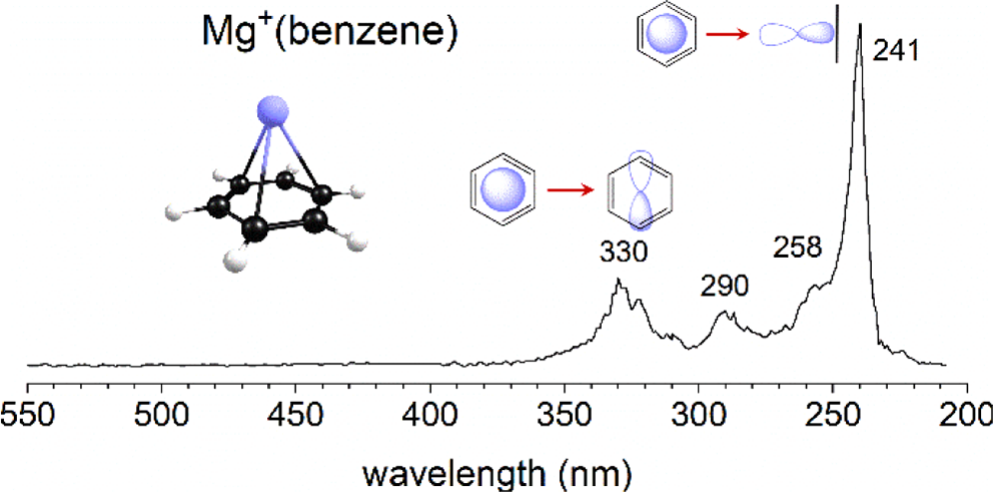

Tunable laser photodissociation spectroscopy and photofragment imaging experiments are employed to investigate the spectroscopy and dissociation dynamics of the Mg^+^(benzene) ion–molecule complex. When excited with ultraviolet radiation, Mg^+^(benzene) photodissociates efficiently, producing both Mg^+^ and benzene^+^ fragments, with branching ratios depending on the wavelength. The wavelength dependence of these processes are similar, with intense resonances at 330 and 241 nm and weaker features at 290 and 258 nm. Comparisons of the experimental spectra to those predicted by computational chemistry at the TD-DFT level allow assignment of these to metal ion-based (330 and 241 nm), charge-transfer (290 nm), and benzene-based (258 nm) transitions. However, the observation of the benzene cation fragment at all wavelengths, which can only result from charge-transfer, indicates unanticipated excited state dynamics. Spectroscopy experiments are complemented by photofragment imaging to investigate these dynamics. The high kinetic energy release indicates that multiphoton absorption based on the intense atomic resonances is responsible at least in part for the dissociation processes.

## Introduction

Metal ion-benzene complexes and their sandwiches provide prototypical examples of organometallic bonding and cation-π interactions.^1−12^ Mass spectrometry has investigated the intrinsic bonding properties of these systems in the absence of solvents or counterions. Various experimental studies have explored the reactions, thermochemistry, and photochemistry of these ions, whereas spectroscopy and computational chemistry have investigated their electronic structure and bonding.^13−59^ Early photochemistry experiments revealed the importance of charge-transfer processes in excited electronic states of these systems.^14−16,42^ Photofragment imaging studies have since explored the energetics and dynamics of these processes,^55,57−59^ but there are only limited studies of the corresponding spectroscopy.^59^ In the present system, we investigate both the electronic spectroscopy and corresponding charge-transfer photodissociation dynamics of the Mg^+^(benzene) complex.

Metal ion-benzene complexes have been studied with a variety of experimental methods to determine their dissociation energies, and data are available for many M^+^(benzene)_1,2_ complexes.^18,28,32,35,36,56^ Collision-induced dissociation (CID) has been employed for many of these measurements, complemented by computational chemistry. Recent experiments have employed tunable laser photodissociation thresholds and photofragment imaging experiments to investigate this thermochemistry,^55,57−59^ validating the results of CID in several systems.

The optical properties and photochemistry of metal ion-benzene systems are often characterized by strong charge-transfer resonances.^14−16,42,59^ The ground state of these systems has the charge localized on the metal ion because the ionization energy of the metal is usually much lower than that of benzene. However, optical excitation in the visible or UV region often leads to photodissociation, producing a significant yield of the benzene cation photofragment. This process has been investigated with photodissociation channels for various excitation wavelengths, with photofragment imaging measurements, and with tunable laser photodissociation spectroscopy.^14−16,42,55,57−59^ In addition to charge transfer, another possibility in the spectroscopy of metal ion-benzene systems is HOMO–LUMO excitation on the benzene. The well-known π → π* transition in benzene occurs at 260 nm in the isolated molecule, but this may shift upon cation-π bonding with metal ions.^59^ Another aspect of the study for Mg^+^(benzene) is that the Mg^+^ ion has intense 3s → 3p (^2^S → ^2^P_1/2,3/2_) atomic resonances at 35669/35761 cm^–1^ (280.4 and 279.6 nm).^60^ The atomic transitions are shifted and split into multiplets by ligand interactions, and this has been studied in the electronic spectroscopy of several Mg^+^(L) systems.^61−70^ Mg^+^(benzene) provides the opportunity to study a system where all of these optical phenomena may be active in the spectroscopy of the same ion.

## Experimental Section

Ion–molecule complexes of the form Mg^+^(benzene)_n_ were produced by laser vaporization^71^ in a pulsed supersonic expansion of argon containing benzene vapor at its ambient concentration above the room temperature liquid. Ions produced in this way are typically believed to have rotational temperatures of 10–50 K.^71^ The ions were analyzed and mass selected for study with a reflectron time-of-flight mass spectrometer designed for photodissociation experiments.^72,73^ Mass selection is accomplished with pulsed deflection plates in the first flight tube of the reflectron instrument, photodissociation takes place at the turning point in the reflectron field, and fragment mass analysis is accomplished using the flight time through a second drift-tube section. Tunable UV–visible radiation for threshold spectroscopy experiments was provided by a Nd:YAG-pumped optical parametric oscillator (OPO) laser system (Continuum Horizon II; line width 5–7 cm^–1^; 1.0 mJ/pulse energy; unfocused; 4 mm diameter beam spot). The yield of the fragment mass recorded versus the photon energy provides the photodissociation spectrum. The laser step size for survey scans is 1 nm; higher resolution scans used 0.01 nm steps.

Photofragment imaging studies were conducted using our selected-ion velocity-map imaging (SI-VMI) instrument.^55,57,58^ In this device, ions are selected by their flight time though a linear time-of-flight instrument and then transmitted into an imaging flight tube where photodissociation occurs. The photodissociation laser is either the UV–visible OPO indicated above or a Nd:YAG (Spectra Physics GCR-170) operating on the second, third or fourth harmonic wavelengths (532, 355, or 266 nm) using the same pulse energies employed for the spectroscopy (i.e., about 1.0 mJ/pulse, unfocused). Photofragment ions are reaccelerated using a series of electrostatic lenses designed for velocity map imaging (VMI),^74−79^ and detected using the DC-slice imaging method.^80^ To achieve slicing, the dual MCP/P-47 phosphor detector (Beam Imaging Solutions BOS-75) is activated in a narrow time window with a fast rise-time high voltage pulser (DEI PVX-4140), allowing fragment ions in the central ∼90 ns of the arrival-time distribution to be detected. Images are collected using a CCD camera (Edmund Optics), averaging over several hundred thousand laser shots. Images are processed with the NuACQ and BasisFit software.^81^ Calibration was accomplished by measuring the image of Ar^+^ from the photodissociation of Ar_2_^+^ using the same instrument settings.^82^ The design for this instrument using photofragment imaging of jet-cooled ions that are mass-selected is unique to our lab,^55,57^ but similar instruments have recently been reported by other groups.^83−86^

Computational studies on the magnesium cation-benzene complexes were carried out with the Gaussian16 program package,^87^ using DFT with the def2-TZVP basis set.^88^ Calculations were performed using several different functionals (B3LYP, M06-L,^89,90^ MN15-L).^91^ All energetics, i.e., dissociation energies, were zero-point corrected using harmonic frequencies, and all structures were confirmed to be minima. Electronic spectra were predicted using time-dependent density functional theory (TD-DFT) using the B3LYP functional. Transition frequencies represent vertical transitions from the optimized ground state with no Franck–Condon analysis, and the band positions are used without any electronic scaling factor.

## Results and Discussion

**Figure 1:**
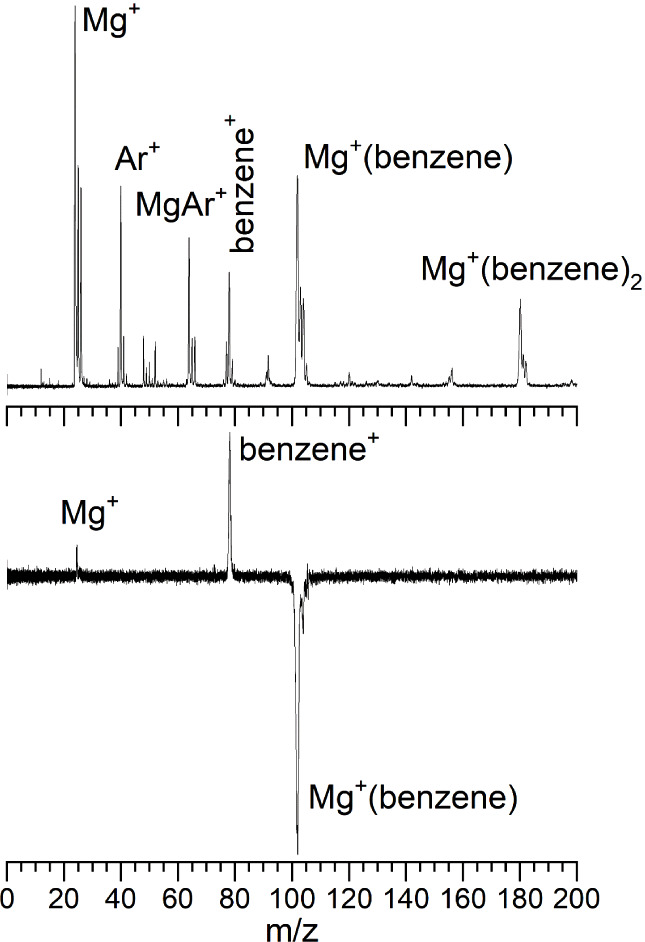
Mass spectrum of Mg^+^(benzene)_n_ ions and the photodissociation mass spectrum of Mg^+^(benzene) at 355 nm.

Laser vaporization produces a distribution of cation-molecular complexes of the form Mg^+^(benzene)_n_. A typical mass spectrum is shown in Figure 1, where we also present the photodissociation mass spectrum produced when the Mg^+^(benzene) ion is mass-selected and excited at 355 nm. The photodissociation is presented as a difference spectrum, in which the intensity of the Mg^+^(benzene) ion without laser excitation is subtracted from that with laser excitation. The depletion of the parent ion is shown as a negative peak and the photofragments are presented as positive peaks. As shown, the photofragments include both the Mg^+^ ion, resulting from elimination of a neutral benzene molecule, and the benzene^+^ cation, resulting from a photoinduced charge-transfer dissociation process. The latter process is evident because the ground state of the Mg^+^(benzene) ion has the charge localized mostly on the metal. This is expected because the ionization energy of Mg is 7.65 eV^92^ and that for benzene is 9.24 eV,^93^ and it is confirmed by the collisional dissociation of this ion which occurs in the ground electronic state and produces only the Mg^+^ fragment.^28,35^ These photodissociation products from Mg^+^(benzene) have been reported previously by our group.^16^

### Photodissociation Spectroscopy

**Figure 2:**
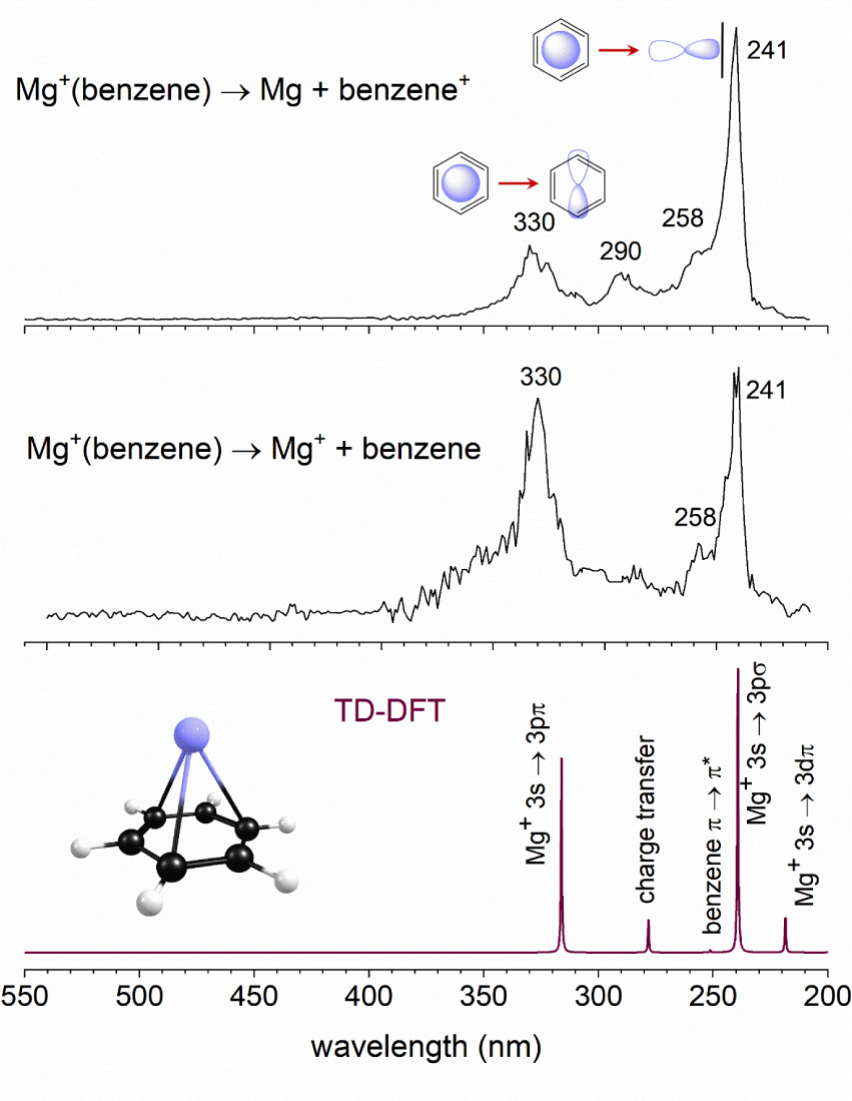
Photodissociation spectrum of Mg^+^(benzene) in the benzene cation fragment ion channel (top) and that in the Mg^+^ fragment ion channel (middle) compared to the electronic absorption spectra predicted by TD-DFT using the B3LYP functional. The intensity of the signal in the Mg^+^ channel (middle) was multiplied by a factor of 10 for this comparison.

Figure 2 shows the wavelength dependence of the photodissociation in the range of 550 to 200 nm compared to the predictions of time-dependent density functional theory (TD-DFT). The spectrum measured in the benzene cation fragment channel is presented in the upper frame, whereas that for the Mg^+^ fragment ion is in the middle frame. The Mg^+^ fragment spectrum was multiplied by a factor of 10× to compare to the spectrum in the much more intense benzene^+^ channel. There are two broad, intense resonances located at 330 and 241 nm, with additional weaker structure in these spectra. Except for the intensity of the 330 nm feature, the patterns in these spectra are essentially the same for the benzene cation and the Mg^+^ cation fragments, although the benzene cation signal is much more intense throughout these spectra. The overall efficiency of photodissociation throughout these spectra is quite high. There is some uncertainty in the overlap of the laser with the ion beam, but the cross section for photodissociation on the 241 nm resonance is determined to be approximately 10^–16^ cm^2^. Previous studies of Mg^+^(L) complexes found structured spectra with vibrationally and rotationally resolved features.^61−70^ However, higher resolution scans of the bands here found no resolved structure. It is not clear whether this lack of structure is caused by a high density of overlapping vibronic structure, or from lifetime broadening from rapid radiationless transitions.

To further investigate this system, density functional theory (DFT) computations were carried out on the Mg^+^(benzene) ion in its ground state, and time-dependent density functional theory (TD-DFT) calculations were conducted for transitions to excited electronic states. The results of these computations are presented in Table 1. Because of the 3s^1^ valence configuration on Mg^+^ and the high energy to move electrons from the 2p level of the metal ion or to excite benzene, all the considered states are doublets (i.e., *m* = 2). The ground state is predicted to have the metal ion in the symmetric position on the 6-fold axis located 2.29 Å above the benzene ring. The benzene ring maintains its planarity in this complex, resulting in C_6v_ symmetry. According to theory, the bond energy between the Mg^+^ ion and the benzene is 31.1 kcal/mol at the DFT/B3LYP level, and this varies somewhat for other functionals as shown in Table 1. The computational energetics can be compared to the experimental value for the dissociation energy of 32.0 ± 2.4 kcal/mol determined by Armentrout and coworkers using collision-induced dissociation.^28^

**Table 1:** Computed Energetics for Mg^+^(benzene) Complexes Employing Different DFT Functionals^a^

	B3LYP	M06-L	MN15-L	Exp.
Energies				
Benzene	–232.237	–232.194	–232.022	
Mg^+ 2^S	–199.807	–199.777	–199.729	
Mg^+^(benzene)	–432.094	–432.025	–431.815	
Dissociation Energies				
Mg^+^(benzene)	31.1	34	39.6	32.0 ± 2.4

a Energies (zero-point corrected) are in Hartrees, except for dissociation energies.

**Figure 3:**
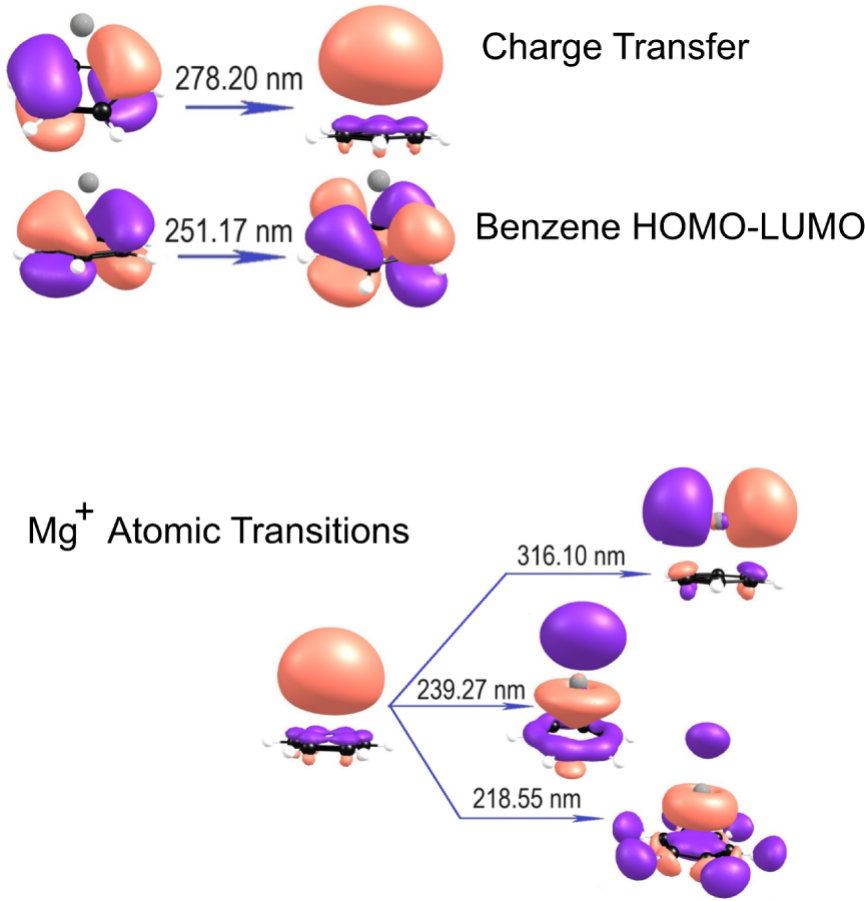
Orbitals involved in the various electronic transitions for the Mg^+^(benzene) complex.

The TD-DFT computations for the absorption spectra of the Mg^+^(benzene) ion reveal the energetics of transitions associated with metal ion-based resonances, charge-transfer resonances, and the HOMO–LUMO transition on the benzene ligand. The results of these calculations are presented in Table 2. The predicted absorption spectrum (lower frame, Figure 2) reproduces the positions and relative intensities of these spectra remarkably well. Because of this agreement, we are confident enough to use theory to assign the transitions in the spectrum. The band at 330 nm is therefore assigned to the Mg^+^ 3s → 3pπ atomic-based transition. The 3pπ configuration has the *p* orbital in the Mg^+^ excited state lying parallel against the plane of benzene. There are doubly degenerate versions of this excited state for x and y orientations of this orbital. The 290 nm band is assigned to the Mg^+^(benzene) → Mg(benzene^+^) charge-transfer transition. The weak shoulder at 258 nm is the π → π* HOMO–LUMO transition on the benzene ligand. The band at 241 nm is assigned to the Mg^+^ 3s → 3pσ atomic-based transition. This configuration has the *p* orbital in the Mg^+^ excited state pointed perpendicular into the plane of benzene. Finally, a weak band is predicted near 220 nm, and a slight hint of a signal is detected here. This arises from the Mg^+^ 3s → 3dπ atomic-based transition in which an excited *d* orbital of Mg^+^ lies parallel to the face of benzene. This transition is forbidden on the Mg^+^ atomic ion, but becomes weakly allowed in the reduced symmetry of the complex. Although it is generally expected that charge-transfer transitions should be intense, in this case the atomic-based resonances on the Mg^+^ lead to the most intense resonances. This is not too surprising because the 3s → 3p (^2^S → ^2^P_1/2,3/2_) atomic transition is extremely strong, with an Einstein *A* coefficient of 2.6 × 10^8^ s^–1^ for each of the spin–orbit components.^60^ The orbitals and transitions involved in these spectra are depicted in the form of the starting and ending molecular orbitals in Figure 3.

**Table 2:** Wavelengths, Intensities, and Orbital Characters of the Electronic Transitions of Mg^+^(benzene) Determined from TD-DFT Computations Using the B3LYP Functional

Transition Wavelength	Oscillator Strength, f	Transition Character
316.1	0.1134	Mg^+^ 3s → 3p (p orb. || to benzene)
316.09	0.1134	Mg^+^ 3s → 3p (p orb. || to benzene)
278.2	0.0203	benzene HOMO → Mg^+^ (3s)
278.2	0.0203	benzene HOMO → Mg^+^ (3s)
251.17	0.0015	benzene HOMO → LUMO
251.17	0.0015	benzene HOMO → LUMO
239.27	0.3539	Mg^+^ 3s → 3p (p orb. ⊥ to benzene)
218.55	0.0469	Mg^+^ 3s → 3d (d orb. || to benzene)

**Figure 4:**
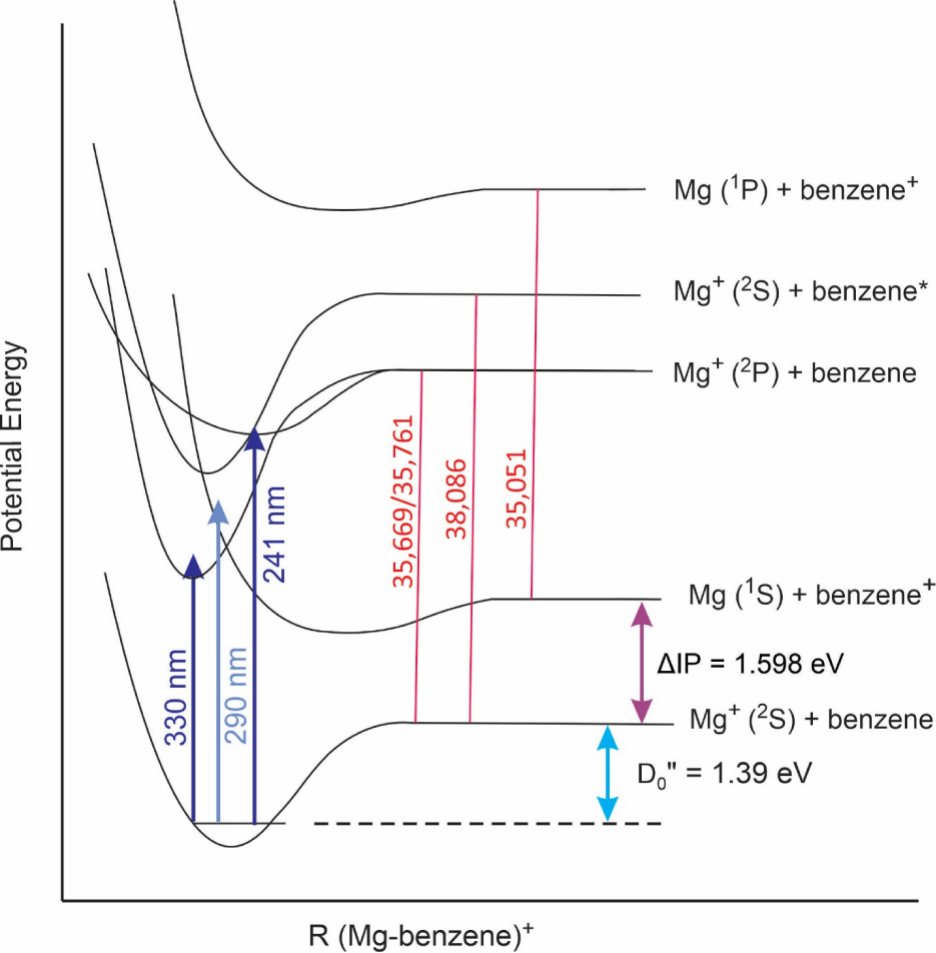
Schematic potential energy curves for the ground and electronically excited states of the Mg^+^(benzene) cation.

Further insight is provided with schematic potential energy curves for the ground and excited states of Mg^+^(benzene), as shown in Figure 4. To construct this figure, we used the experimental ground state dissociation energy from CID experiments (32.0 kcal/mol; 1.39 eV),^28^ and the known atomic and molecular states for Mg^+^ and benzene for the asymptotic energies. The depths of the bound molecular complex excited states were estimated based on the considerations discussed below. The first excited asymptotic state is the Mg + benzene^+^ charge-transfer state. As discussed previously for these kinds of states, the excited potential well is expected to be rather shallow. The electrostatic interaction between a molecule with delocalized charge interacting with a neutral atom should be weaker than that in the ground state, which has an atomic cation interacting with a large polarizable molecule. A similar shallow well would be expected for higher energy charge-transfer states (e.g., Mg ^1^P + benzene^+^). The vertical transition from the ground state should access the repulsive wall of this potential. It is therefore not surprising to observe the benzene^+^ cation photofragment when this excited state is produced. The minimum energy required for charge transfer is the interval between the bottom of the ground state potential and the charge-transfer asymptote, and this is about 2.99 eV. Therefore this process is energetically possible at wavelengths shorter than about 415 nm. However, the transition is observed experimentally at 290 nm. This suggests that the vertical transition lands on the repulsive wall of the excited potential well above the minimum energy for the transition. The exact excited state potential is not known, but would of course be interesting to investigate. Unfortunately, calculating the excited state potential would be difficult because of the well-known problems that density functional theory has with excited states and charge transfer in particular. In the present system, the charge-transfer state overlaps with other transitions associated with the metal ion, and this causes additional complications for theory on excited states.

The second excited state asymptote is that for the Mg^+^ (^2^P_1/2,3/2_) + benzene, which is the first excited state for the magnesium cation. The atomic 3s → 3p (^2^S → ^2^P_1/2,3/2_) transition has an energy of 35,669/35,761 cm^–1^ for the two spin orbit levels.^60^ This atomic transition is expected to shift to new energies for the molecular transitions in the ion–molecule complex because of the interactions between the metal orbitals and the benzene ligand. The 3pπ configuration has the *p* orbital in the Mg^+^ excited state lying parallel against the plane of benzene. Because the *p* orbital has a node on the center line, this configuration exposes the Mg^+^ core which resembles the Mg^2+^ ion to the π cloud of benzene, which is a favorable electrostatic interaction. The excited state well depth for this configuration should be much greater than that for the ground state, which has the core shielded by the singly occupied 3s orbital. The transition from the ground Mg^+^ (^2^S) + benzene state to the excited Mg^+^ (3pπ) + benzene excited state therefore is strongly shifted to lower energy than the atomic transition. The red shift is caused by the difference between the binding energies of the excited state and the ground state. Without vibrational structure and assignments we cannot determine this exactly, but the differential binding energy, determined from the position of the 330 nm band relative to the atomic transition, is about 5,000 cm^–1^. Using the known ground state well depth (32.0 kcal/mol; 11,200 cm^–1^) and this energy difference, we can infer that the Mg^+^ (3pπ) + benzene excited state is bound by about 16,000 cm^–1^ (1.98 eV; 45.7 kcal/mol). This determines the well depth for this excited state in Figure 3. The same kind of logic can be applied to the Mg^+^ (3pσ) + benzene excited state. In this state, the metal ion 3p orbital is pointing into the benzene π system, shielding the ion-induced dipole interaction even more effectively than the 3s orbital in the ground state. The transition from the ground Mg^+^ (3sσ) + benzene state to the excited Mg^+^ (3pσ) + benzene excited state occurs at 241 nm, which is strongly shifted to higher energy than the atomic transition. Again, this shift is associated with the differential binding energies between the ground and excited state. The excited state is therefore about 6000 cm^–1^ less strongly bound than the ground state, suggesting that its well depth is about 5000 cm^–1^ (0.62 eV; 14.3 kcal/mol). This determines the well depth for this excited state potential in Figure 3.

The other transition observed in the spectrum is that for the HOMO–LUMO excitation on the benzene ligand. As shown in the level diagram, the energy at the asymptote is that for the transition in the isolated benzene molecule, which is 38,086 cm^–1^.^94^ If the benzene ligand is unperturbed, the transition in the Mg^+^(benzene) complex should occur at about this same energy, or at 262.5 nm. Instead, we observe it at 258 nm, which is a slight blue shift. The presence of the nearby cation shifts this transition to slightly higher energy, either by stabilizing the ground state or destabilizing the excited state.

Although the nature of the transitions in the spectrum is understandable, the photochemical dynamics are somewhat puzzling. As shown in the figure, excitation at 330, 258, and 241 nm all land in bound excited states, which should not dissociate directly. Nevertheless, excitation at all of these wavelengths causes efficient photodissociation producing primarily the benzene cation. Production of the benzene cation indicates that the final separation of Mg and benzene^+^ occurs on the charge-transfer potential surface. However, the initial excitation of these other excited states does not land on this potential. Some sort of curve crossing must take place that leads to dissociation on this excited state. This could take place from the bound excited state initially populated, or it could occur after absorption of a second (or more) photon in a resonance-enhanced multiphoton photodissociation process.

In previous spectroscopy of Mg^+^(L) complexes, where L = rare gases, CO_2_, N_2_, H_2_O, etc., we measured sharp vibrational and rotational structure in electronic spectra, indicating long-lived bound excited states.^61−70^ We detected all these spectra via photodissociation and interpreted the dissociation to arise from resonance-enhanced multiphoton processes. It seems likely that the same kind of process could be active here because of the intense atomic transitions of Mg^+^. Single-photon resonances are necessary to explain the agreement with the absorption spectrum (band positions and intensities) predicted by theory. The absorption of one or more additional photons could produce higher excited states that then couple with and transfer to the charge-transfer potential before dissociation to the Mg + benzene^+^ products. Rapid electronic relaxation to the lowest energy excited state is consistent with Kasha’s Rule.^95^ Although this is usually assumed to be caused by collisional relaxation in solution, it apparently happens here for isolated molecules. We observed a similar intramolecular relaxation to the lowest energy charge-transfer state in our recent study of the photodissociation spectroscopy of Ag^+^(benzene) and Ag^+^(toluene).^59^

### Photofragment Imaging

**Figure 5:**
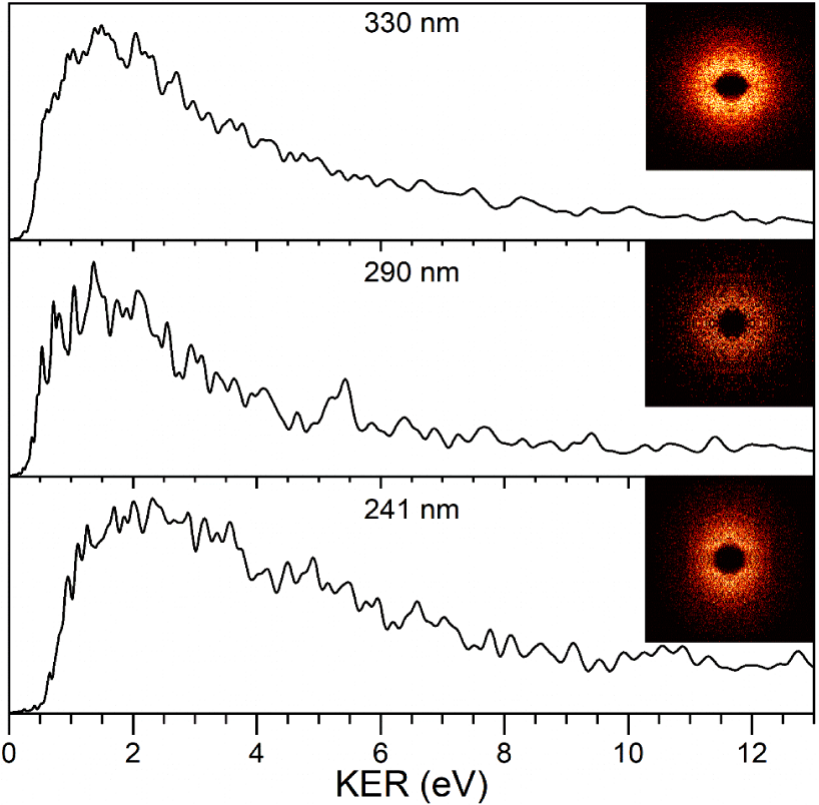
Photofragment images of the benzene^+^ fragment from the photodissociation of Mg^+^(benzene) at 330, 290, and 241 nm and the kinetic energy spectra derived from these images.

To further investigate the dynamics of these photodissociation processes, we have employed photofragment velocity-map imaging. Figure 5 shows the photofragment images for the benzene cation fragment produced by photodissociation on the main resonances in the spectrum at 330, 290, and 241 nm. This figure also shows the kinetic energy release (KER) spectra resulting from the analysis of these images. Each of these images were collected with slicing, and are shown with symmetrization applied. Additional photofragment images collected under a variety of conditions are shown in Figures S5–S11. These include images collected at 532, 355, and 266 nm, which are off-resonance with the main bands in the spectrum, as well as those collected without slicing and the raw images without processing. There is no significant qualitative difference between these different accumulation/processing methods, except that the sliced images show an empty region in the center corresponding to low energy that is not as prominent in the unsliced images. Each image is radially symmetric, with β values in the range of −0.2 to −0.3 (see angular distribution plots in Figures S12–S22). Consistent with this, the KER values do not change when perpendicular vs horizontal laser polarization is employed.

All of these images at different wavelengths are remarkably similar, whether the images are collected on resonances in the spectrum or off the resonant wavelengths. In the kinetic energy spectra, there is no signal at zero energy, a broad distribution of signal in the 1–4 eV range, and signal gradually falling away with a highest KER value in the 6–8 eV range. These KER spectra are essentially invariant to how the images are collected (slicing versus not) or processed (symmetrized versus not). The maximum KER values in each case are much greater than the photon energies used for dissociation, and therefore these images confirm that photodissociation is occurring via some multiphoton absorption in each case. The KER spectra indicate that the dissociation processes produce a broad distribution of internal energy in these ions rather than efficient production of specific kinetic energies. In previous metal ion-benzene complexes studied with photofragment imaging, the maximum KER values produced made it possible to determine upper limits on the bond dissociation energies. In those systems, there were no other excited states in the energy range of the charge transfer state, the kinetic energy release was small and both power dependences and KER values were consistent with single photon processes. However, the present system is more complex. The magnesium ion atomic transitions overlap the charge-transfer transition, making it impossible to know the exact excitation process. The extremely high kinetic energies cannot be explained without multiphoton excitation, and the kinetic energy spectra are broad, producing uncertainties in the exact number of photons absorbed. Laser power dependences are not expected to be informative about the photon numbers because of the uncertain cross sections for different absorption steps including strong resonances.

The level diagram in Figure 4 provides some insight into these various dissociation processes. At 532 nm, the spectrum in Figure 2 shows that there is no efficient absorption. However, photodissociation is observed at this wavelength when the laser power is increased to about 10 mJ/pulse, and photofragment images are obtained (Figure S5). The photon energy (2.33 eV; 18,797 cm^–1^) is greater than the bond energy by about 0.94 eV, which would produce signal on the low energy side of the KER spectrum, and this does not provide enough energy to access the charge-transfer potential, which is needed to produce the benzene cation fragment. This suggests that at least two photons are absorbed. The two-photon energy (4.66 eV) exceeds the bond energy by 3.27 eV, which would produce signal on the high energy side of the KER spectrum, but does not provide enough excess energy to explain the maximum KER value. This indicates that dissociation at 532 nm involves a mixture of two and three-photon absorption processes. At 355 nm (Figure S6), single-photon absorption is 2.10 eV exothermic, whereas two-photon absorption is 5.59 eV exothermic. Thus, a combination of one and two-photon absorption can explain most of the dissociation signal, with a small amount of three-photon absorption needed to explain the highest KER. At 330 nm (Figure 5), single photon absorption is 2.37 eV exothermic, whereas two-photon absorption is 6.13 eV exothermic. Again, single-photon dissociation is possible, but a significant amount of two-photon dissociation is needed to explain the maximum KER. Similar arguments apply to the photodissociation at 290 (Figure 5), 266 (Figure S9) and 241 nm (Figure 5). It should be noted that 290 nm excitation into the charge-transfer resonance could produce the benzene cation product directly, with low excess energy above the charge-transfer asymptote. However, the photofragment image at this wavelength also has high KER, indicating that multiphoton absorption also occurs in this case.

Because excitation at any of these UV wavelengths provides energy above the charge-transfer asymptote (2.99 eV above the ground state), there is enough energy to access the charge-transfer potential to produce the observed benzene cation fragment. However, the resonance at 290 nm in the spectrum, which is assigned to the charge-transfer transition, occurs well above the threshold energy for charge transfer, presumably because of the Franck–Condon overlap between the ground state and the rapidly rising repulsive wall of this potential. This suggests that energies greater than or equal to the energy at this resonance (4.25 eV) would be required for a direct vertical transition to access this potential. For single-photon excitation, only the 241 nm (5.14 eV) resonance wavelength exceeds this energy, and at least two photons at the other UV wavelengths would be necessary. This Franck–Condon argument and the high KER values detected in all photofragment images, combined with the observation of the benzene cation photofragment at all wavelengths, therefore confirm that multiphoton absorption is essential to explain the absorption spectrum and the dynamics. It is important to note that this multiphoton absorption occurs even though the laser power used for both the spectroscopy and the imaging experiments are quite low (unfocused lasers; pulse energies <1 mJ). This is possible because of the remarkably high absorption cross sections of the transitions on the Mg^+^ and that for the charge transfer. Additionally, one-photon absorptions for the Mg^+^ transitions and the HOMO–LUMO transition on the benzene likely land in bound potentials that do not dissociate directly, and our experiment would not detect this absorption. It is likely that dissociation only occurs after a second photon is absorbed, and this is the signal that we detect.

Another aspect of these photofragment images is their angular distributions. As noted above, and shown in the several images in the Supporting Information, all of these images are essentially isotropic, with no noticeable polarization along the laser axis. This is in marked contrast to previous images involving excitation of metal ion-benzene charge-transfer excited states, which were clearly anisotropic along the laser polarization axis. The lack of polarization indicates either that dissociation is slow on the time scale of molecular rotation, or that the electronic states involved in dissociation have mixed polarization character. The latter scenario likely applies to the present system because of the overlapping and mixed character of the excited states corresponding to charge transfer and metal-based transitions, as well as the multiphoton nature of the dissociation. The total kinetic energies and the angular distribution therefore make sense in the context of multiphoton excitation and the involvement of highly mixed excited states.

## Conclusions

Mg^+^(benzene) provides a fascinating example of ion spectroscopy and photodissociation dynamics. Combined with the predictions of TD-DFT calculations, its spectrum reveals transitions associated with the resonances on the Mg^+^ atomic cation, that for the benzene ligand, and that for the metal-to-ligand charge-transfer. Of these, the intensities of resonances on the magnesium cation outweigh the others, even though charge-transfer transitions are usually expected to be quite intense. To our knowledge, this is the first example of electronic spectroscopy on a metal ion complex exhibiting this variety of chromophores. The absorption process at all wavelengths produces primarily the benzene cation. This observation, combined with the known asymptotic state energies and photofragment imaging data explain these spectra. Absorption in the case of the charge-transfer resonance at 290 nm can access the potential leading to the benzene cation photofragment directly. However, resonances based on the metal ion or the benzene ligand must correspond to initial absorption into bound excited states that do not dissociate directly. Absorption of a second photon is likely at 330 nm, but not required at the 258 (benzene HOMO–LUMO) or 241 nm transitions. In each of these higher energy processes, dissociation requires an electronic curve crossing to access the charge-transfer potential, eventually producing the observed benzene cation photofragment. Production of higher energy excited states via multiphoton absorption explains the high kinetic energy release and the broad distributions of internal energies in the photofragments.

It is important to note that TD-DFT theory using the B3LYP performs reasonably well for this electronic spectrum. Predicted band positions and their intensities match the experimental spectrum without any electronic scaling factor adjustment. Although more sophisticated treatments of these electronic spectra are certainly possible, TD-DFT provides a compelling approach for the prediction and assignment of electronic spectra for metal-containing species. We found similar success with this method in our previous study of Ag^+^(benzene) and Ag^+^(toluene).^59^ However, it should be noted that the excited state dynamics and curve crossings needed to explain the production of the benzene cation product observed here represent challenges not yet routinely accessible by theory for such a metal ion system.

## Supporting Information Available

The full citation for reference 87 and the details of the DFT computations done, including the structures and energetics for each of the cluster ions considered (PDF)
