# Supplementary material for: Photodissociation Spectroscopy and Photofragment Imaging of the Mg+(Benzene) Complex
Source: J Phys Chem A. 2024 Nov 25;128(49):10507–15. doi: 10.1021/acs.jpca.4c05703 (PMC11647887; doi:10.1021/acs.jpca.4c05703)
Supplement: Supplementary file 1 — jp4c05703_si_001.pdf [file jp4c05703_si_001.pdf]

Supporting Information

Photodissociation Spectroscopy and Photofragment Imaging of the Mg^+(benzene) Complex

Jason E. Colley,[1] Nathan J. Dynak,[1] John R. C. Blais,[1] Michael A. Duncan[1]*

[1]Department of Chemistry, University of Georgia, Athens, Georgia 30602, United States

*Email: maduncan@uga.edu

Full citation for Gaussian16 (reference 87):

Frisch, M. J.; Trucks, G. W.; Schlegel, H. B.; Scuseria, G. E.; Robb, M. A.; Cheeseman, J. R.; Scalmani, G.; Barone, V.; Petersson, G. A.; Nakatsuji, H.; Li, X.; Caricato, M.; Marenich, A. V.; Bloino, J.; Janesko, B. G.; Gomperts, R.; Mennucci, B.; Hratchian, H. P.; Ortiz, J. V.; Izmaylov, A. F.; Sonnenberg, J. L.; Williams-Young, D.; Ding, F.; Lipparini, F.; Egidi, F.; Goings, J.; Peng, B.; Petrone, A.; Henderson, T.; Ranasinghe, D.; Zakrzewski, V. G.; Gao, J.; Rega, N.; Zheng, G.; Liang, W.; Hada, M.; Ehara, M.; Toyota, K.; Fukuda, R.; Hasegawa, J.; Ishida, M.; Nakajima, T.; Honda, Y.; Kitao, O.; Nakai, H.; Vreven, T.; Throssell, K.; Montgomery, J. A., Jr.; Peralta, J. E.; Ogliaro, F.; Bearpark, M. J.; Heyd, J. J.; Brothers, E. N.; Kudin, K. N.; Staroverov, V. N.; Keith, T. A.; Kobayashi, R.; Normand, J.; Raghavachari, K.; Rendell, A. P.; Burant, J. C.; Iyengar, S. S.; Tomasi, J.; Cossi, M.; Millam, J. M.; Klene, M.; Adamo, C.; Cammi, R.; Ochterski, J. W.; Martin, R. L.; Morokuma, K.; Farkas, O.; Foresman, J. B.; Fox, D. J. Gaussian 16 (Revision C.01), Gaussian, Inc., Wallingford CT, 2009.

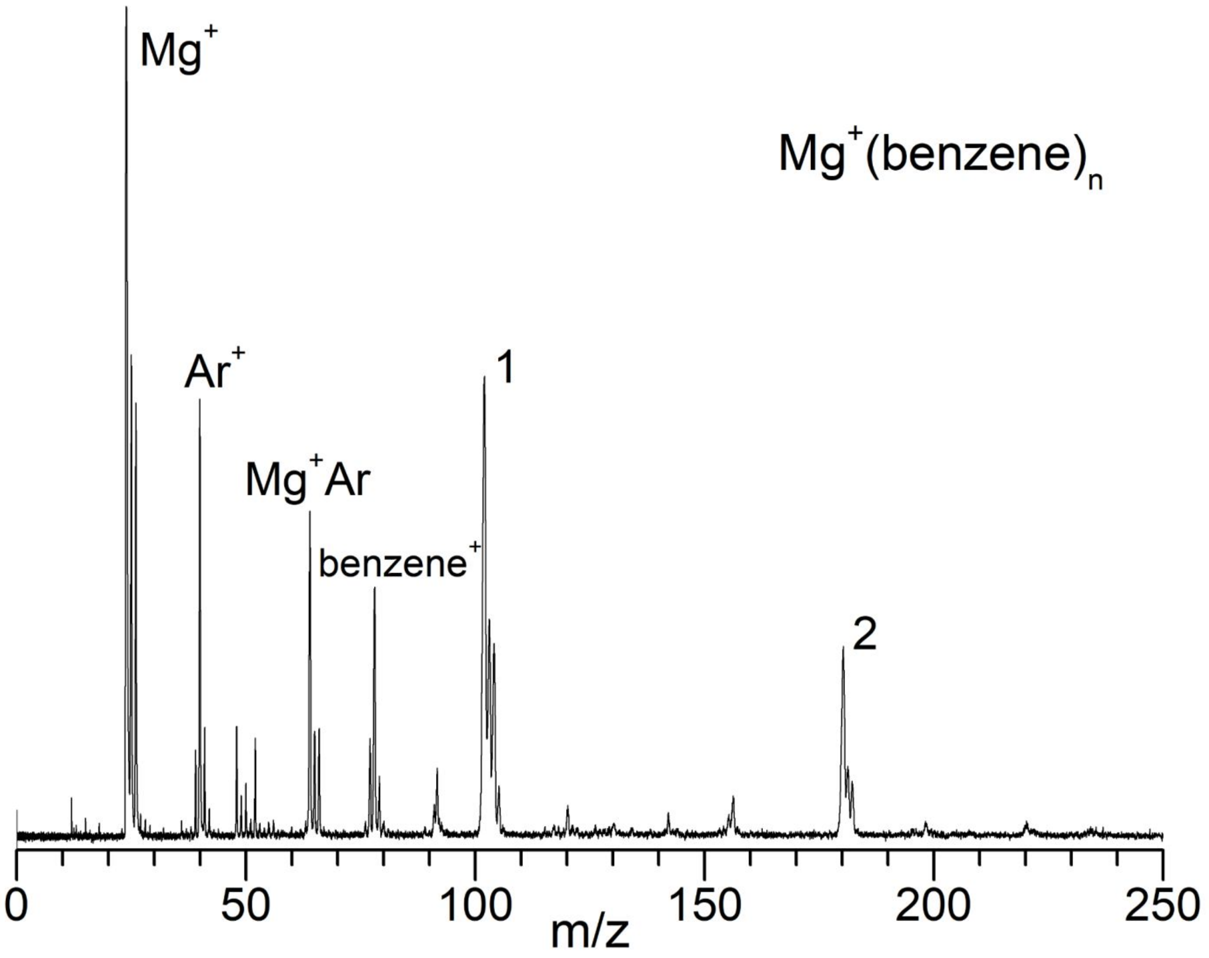

Figure S1. Mass spectrum of magnesium cation-benzene complexes produced by laser vaporization in an argon expansion using a "cutaway" nozzle configuration. A laser pulse energy of 8 mJ/pulse at 355 nm was used for the laser vaporization.

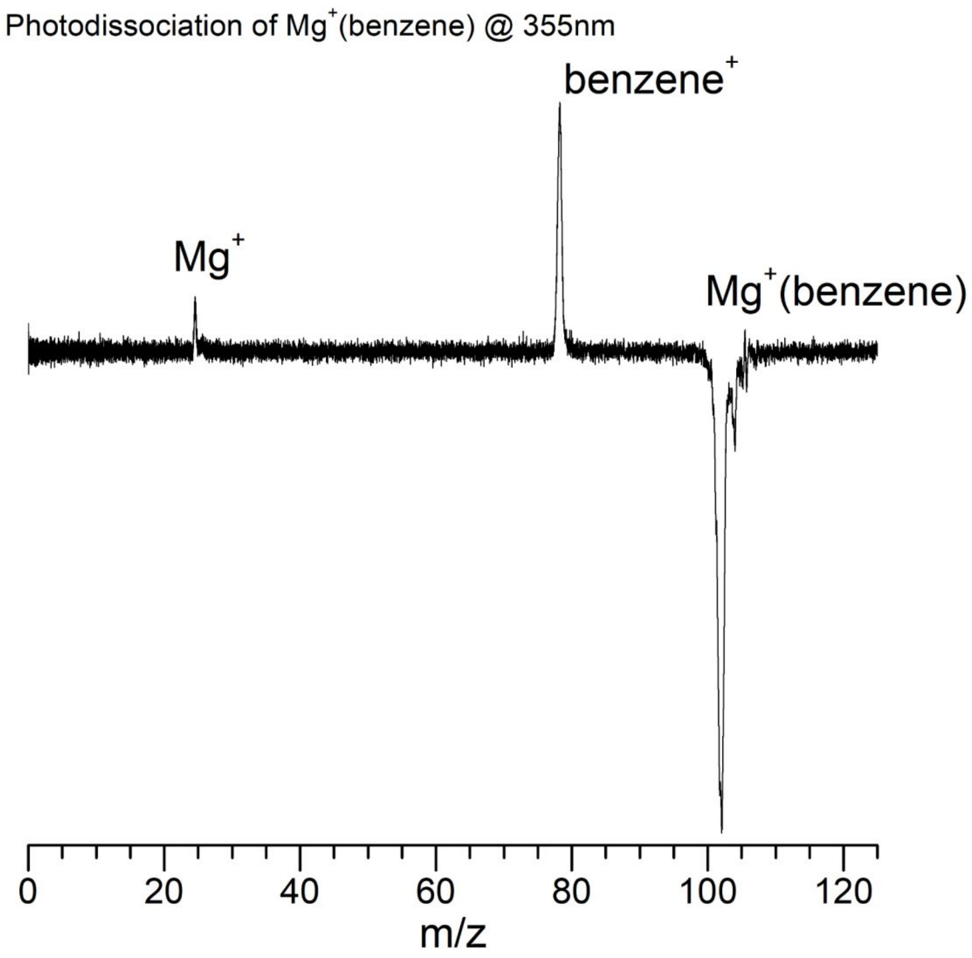

Figure S2. Photodissociation mass spectrum of Mg^+(benzene) at 355 nm which produces both the benzene$^+$ and the Mg^+ photofragments.

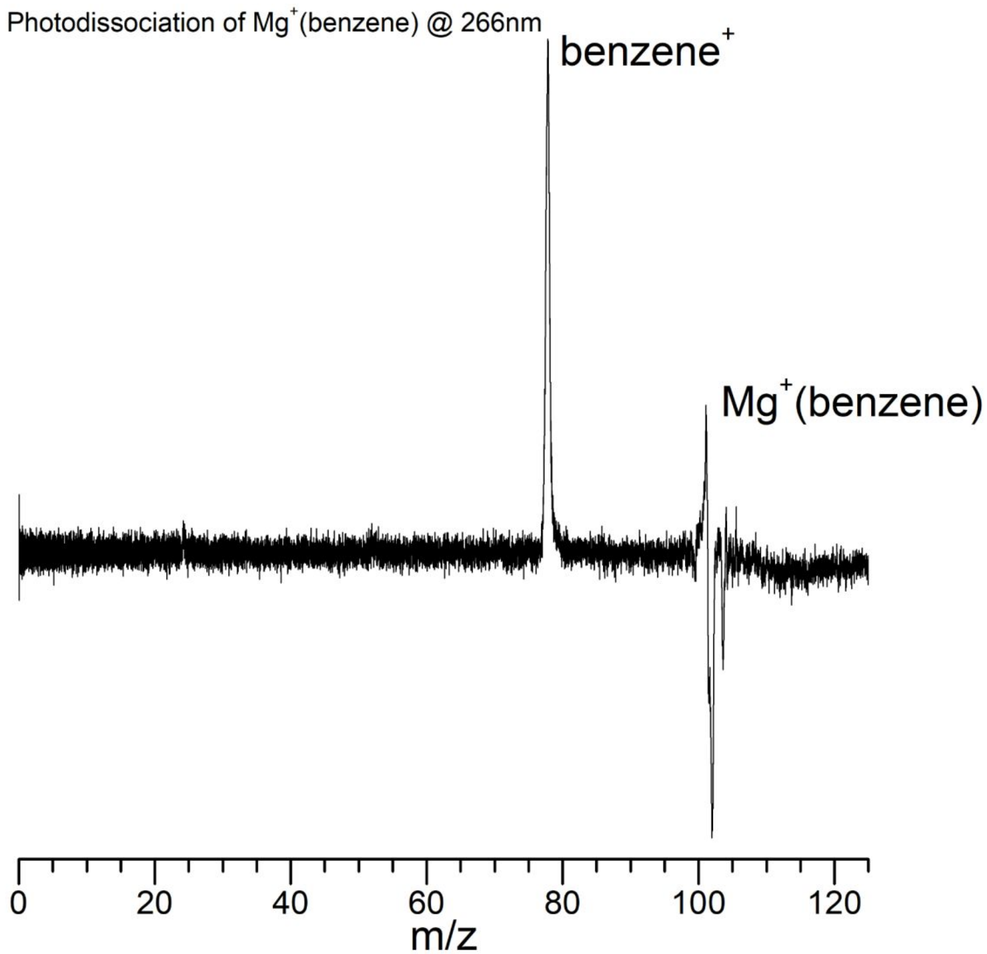

Figure S3. Photodissociation mass spectrum of Mg^+(benzene) at 266 nm, which produces the benzene$^+$ photofragment.

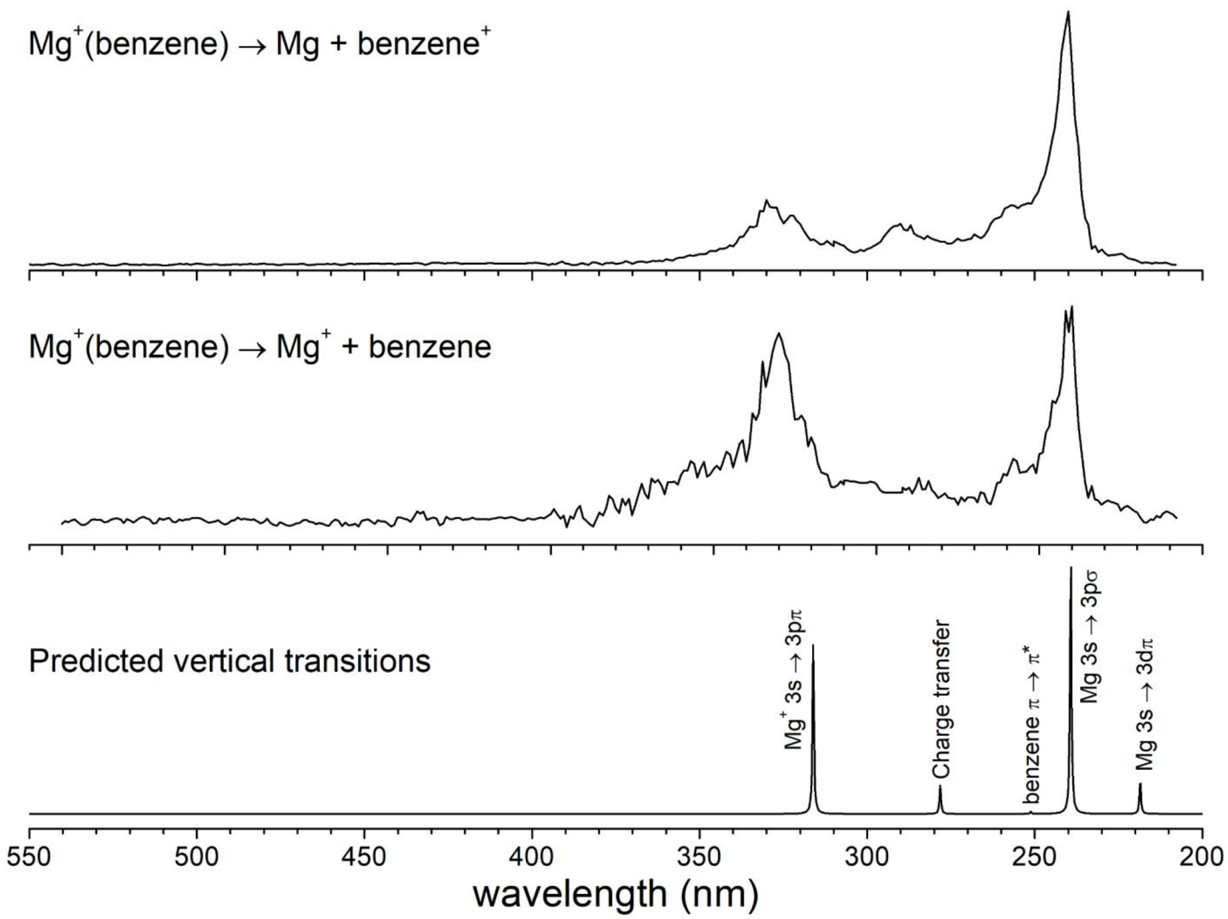

Figure S4. The photodissociation spectrum of Mg^+(benzene) → Mg + $benzene^+$ or Mg^+ + benzene in the 550–208 nm region compared to the predictions of theory for ions in doublet spin state. The Mg^+ channel was multiplied by a factor of 10x for this comparison.

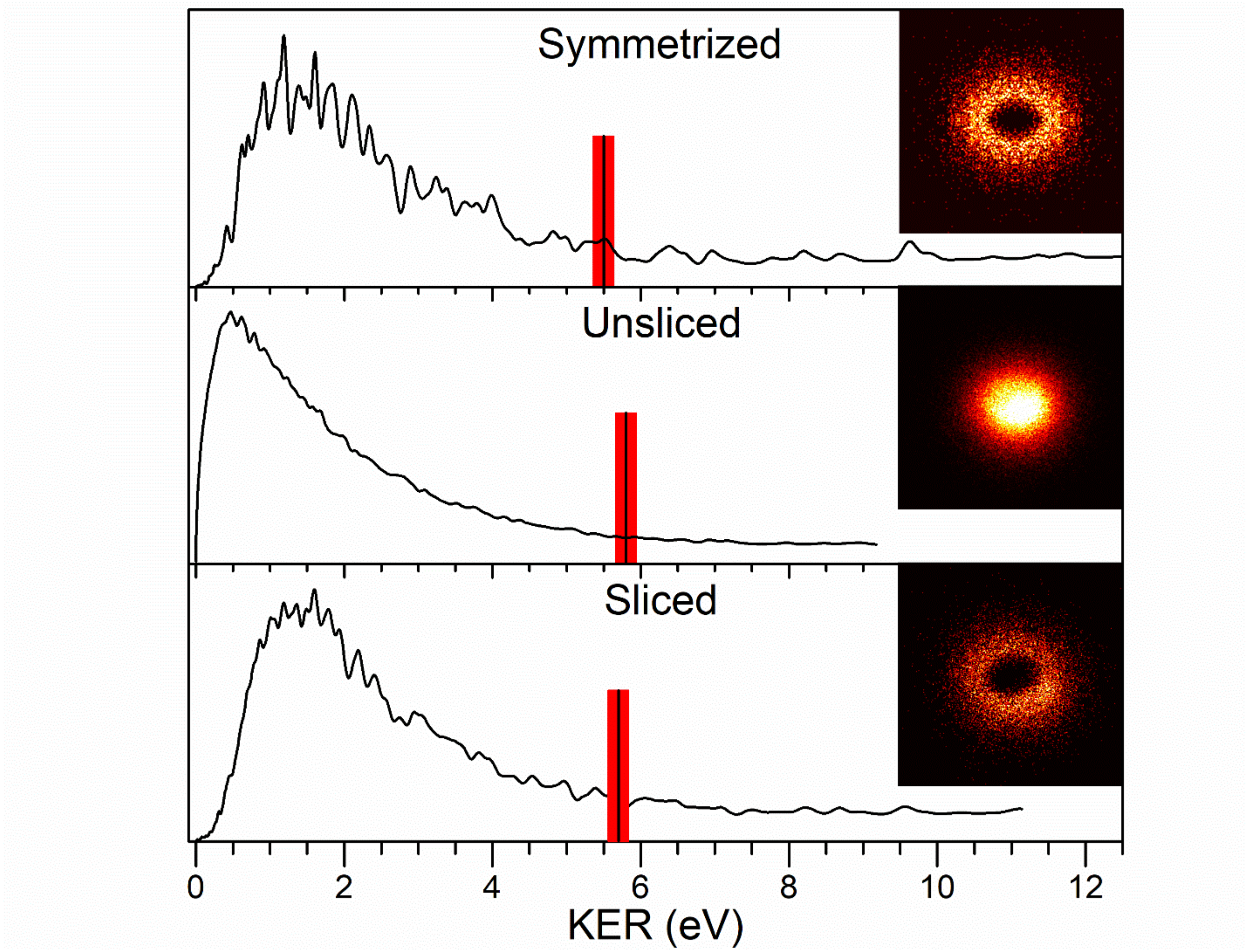

Figure S5. The total energy distribution of the sliced, unsliced, and symmetrized sliced photofragment images of Mg^{+}(benzene) → Mg + $benzene^{+}$ using 532 nm and vertical laser polarization. The black vertical line indicates the assigned value for the maximum kinetic energy, and the red box indicates the width of the instrument resolution.

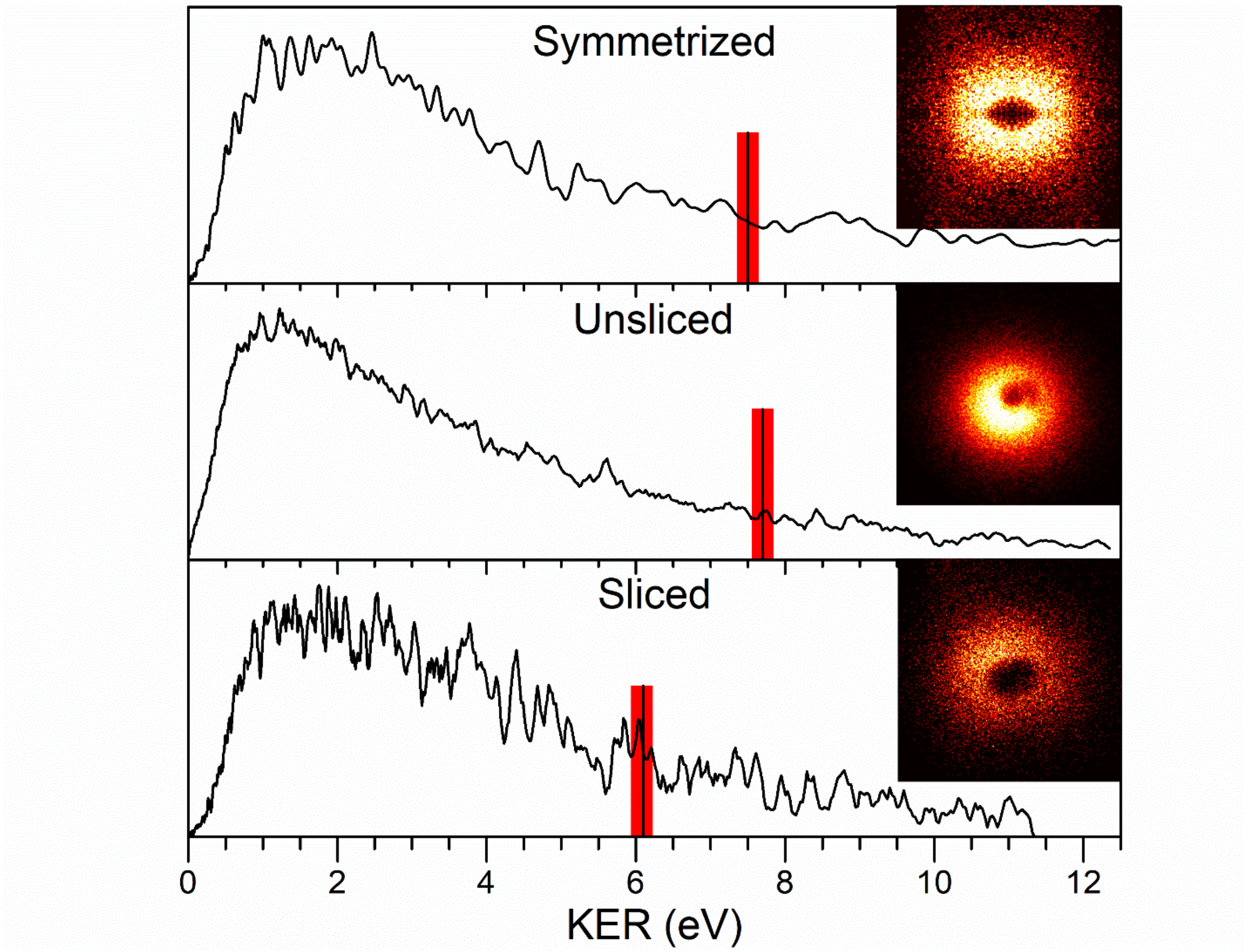

Figure S6. The total energy distribution of the sliced, unsliced, and symmetrized sliced photofragment images of Mg^{+}(benzene) → Mg + benzene^{+} using 355 nm and vertical laser polarization. The black vertical line indicates the assigned value for the maximum kinetic energy, and the red box indicates the width of the instrument resolution.

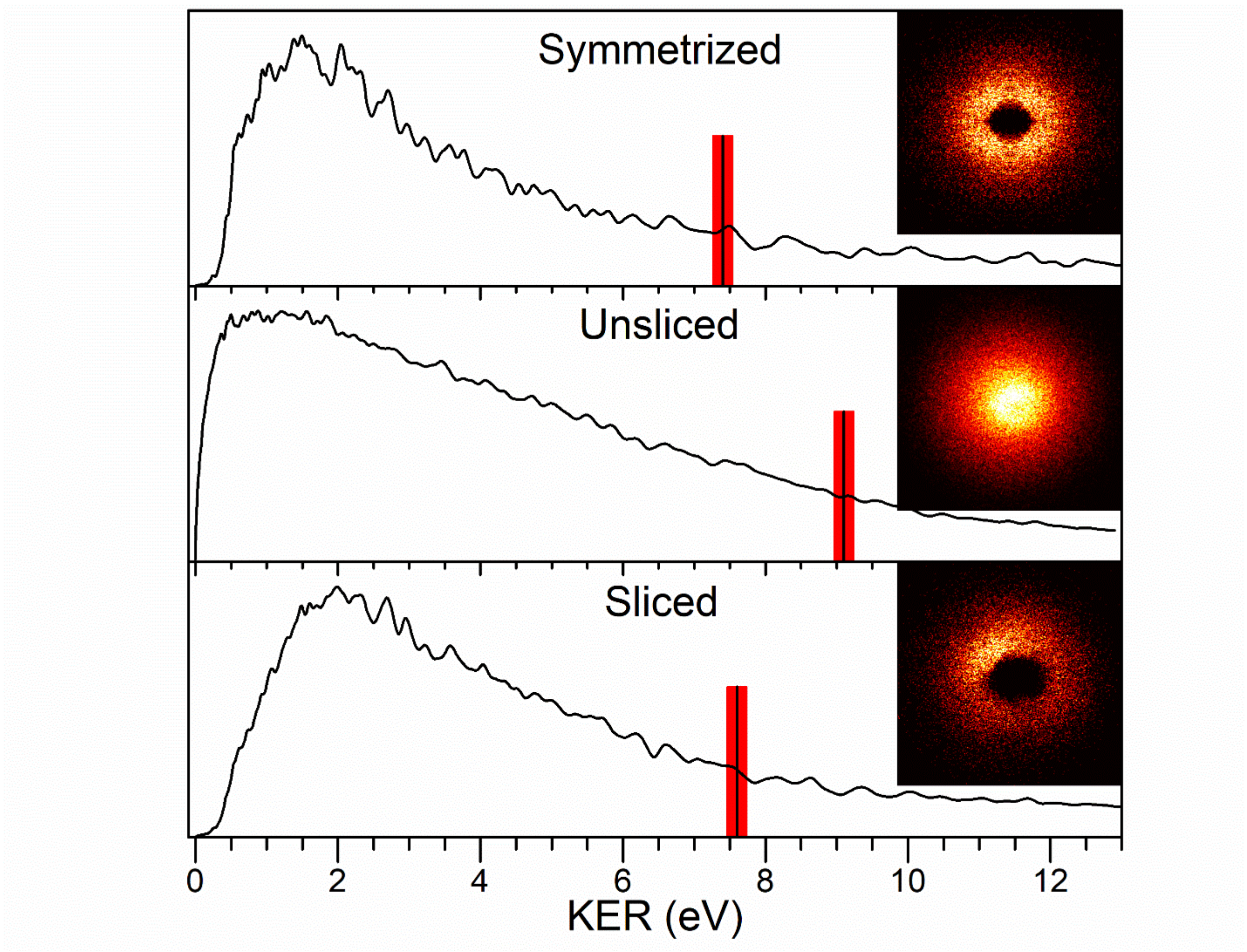

Figure S7. The total energy distribution of the sliced, unsliced, and symmetrized sliced photofragment images of Mg^{+}(benzene) → Mg + $benzene^{+}$ using 330 nm and horizontal laser polarization. The black vertical line indicates the assigned value for the maximum kinetic energy, and the red box indicates the width of the instrument resolution.

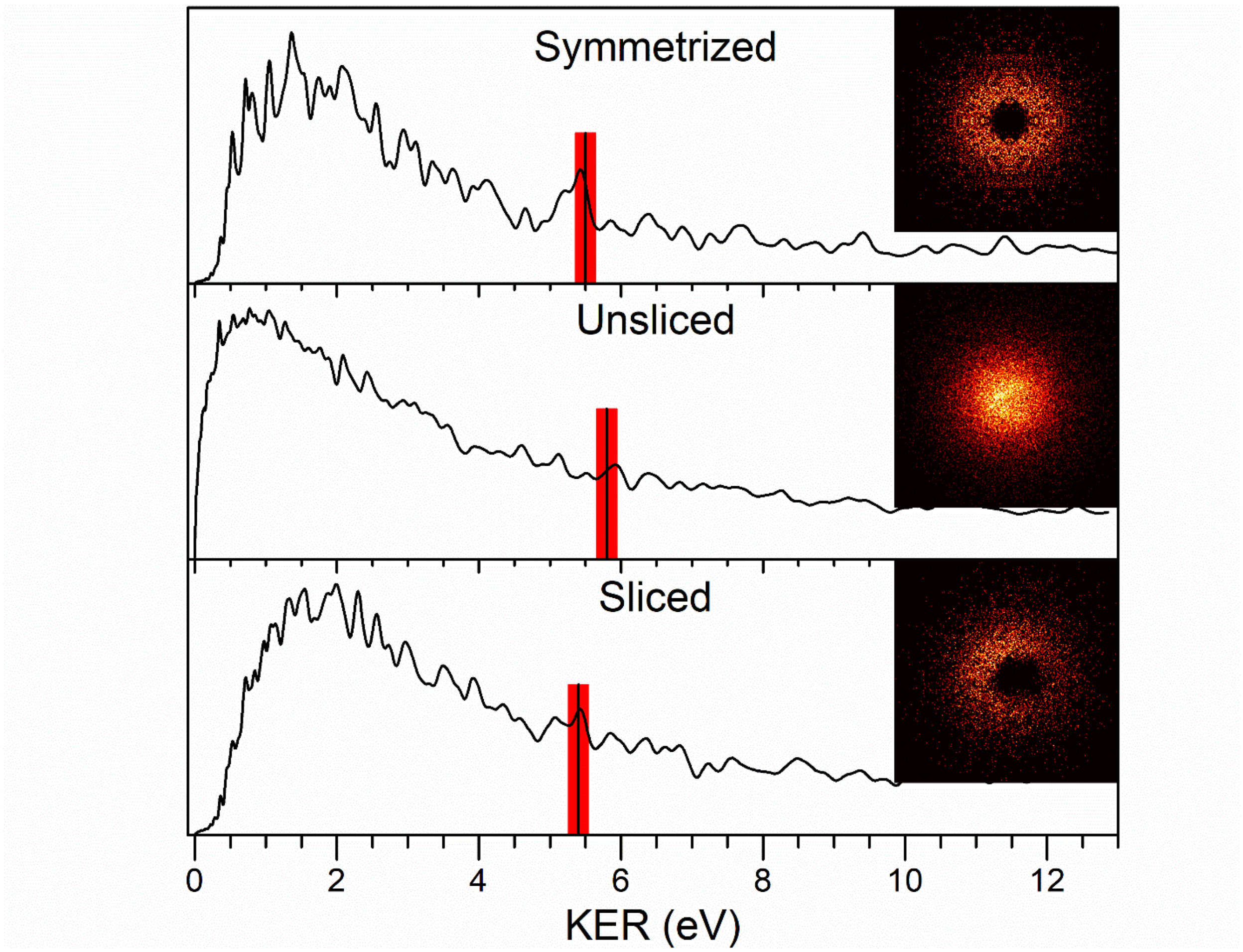

Figure S8. The total energy distribution of the sliced, unsliced, and symmetrized sliced photofragment images of Mg^{+}(benzene) → Mg + $benzene^{+}$ using 290 nm and horizontal laser polarization. The black vertical line indicates the assigned value for the maximum kinetic energy, and the red box indicates the width of the instrument resolution.

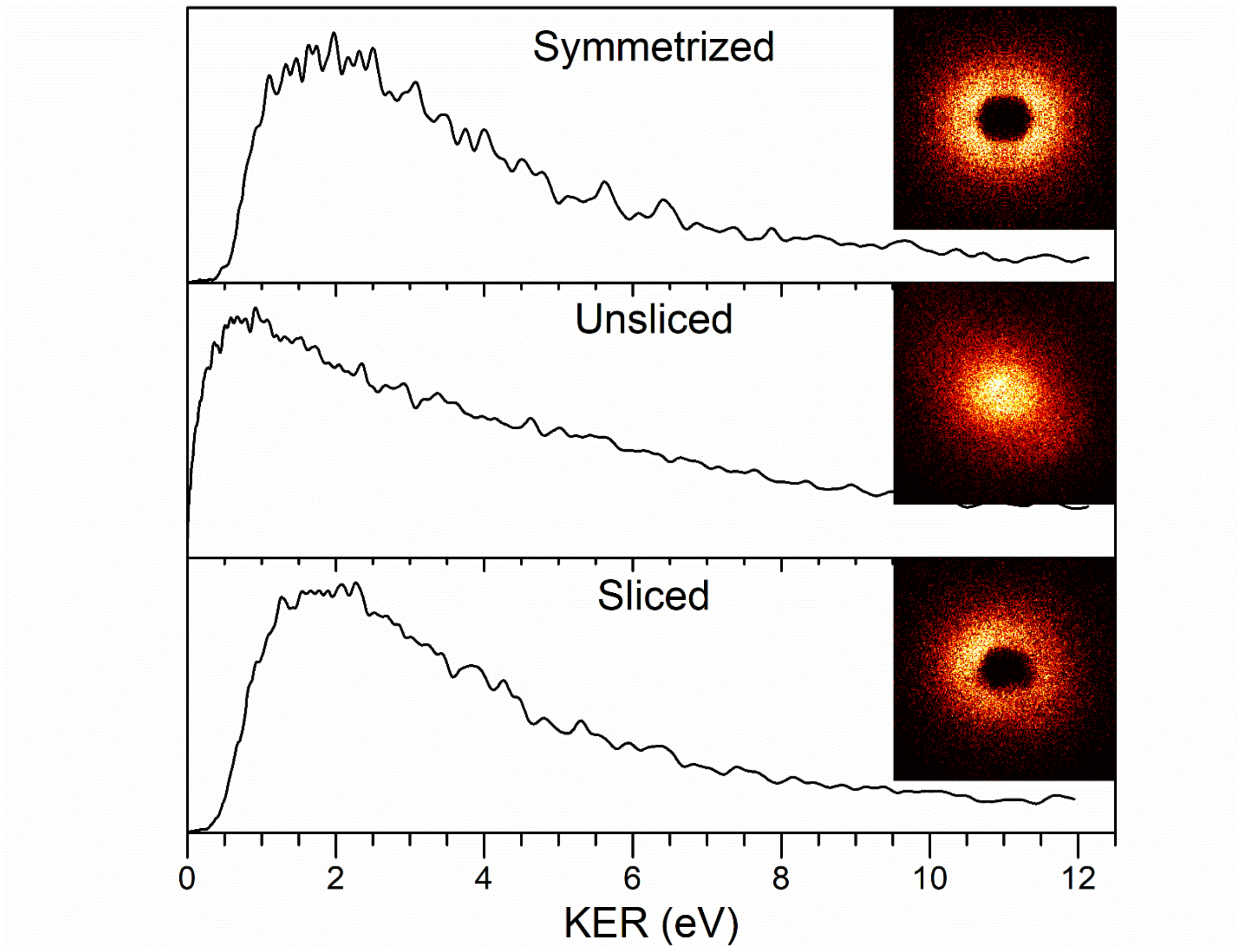

Figure S9. The total energy distribution of the sliced, unsliced, and symmetrized sliced photofragment images of Mg^{+}(benzene) → Mg + $benzene^{+}$ using 290 nm and vertical laser polarization.

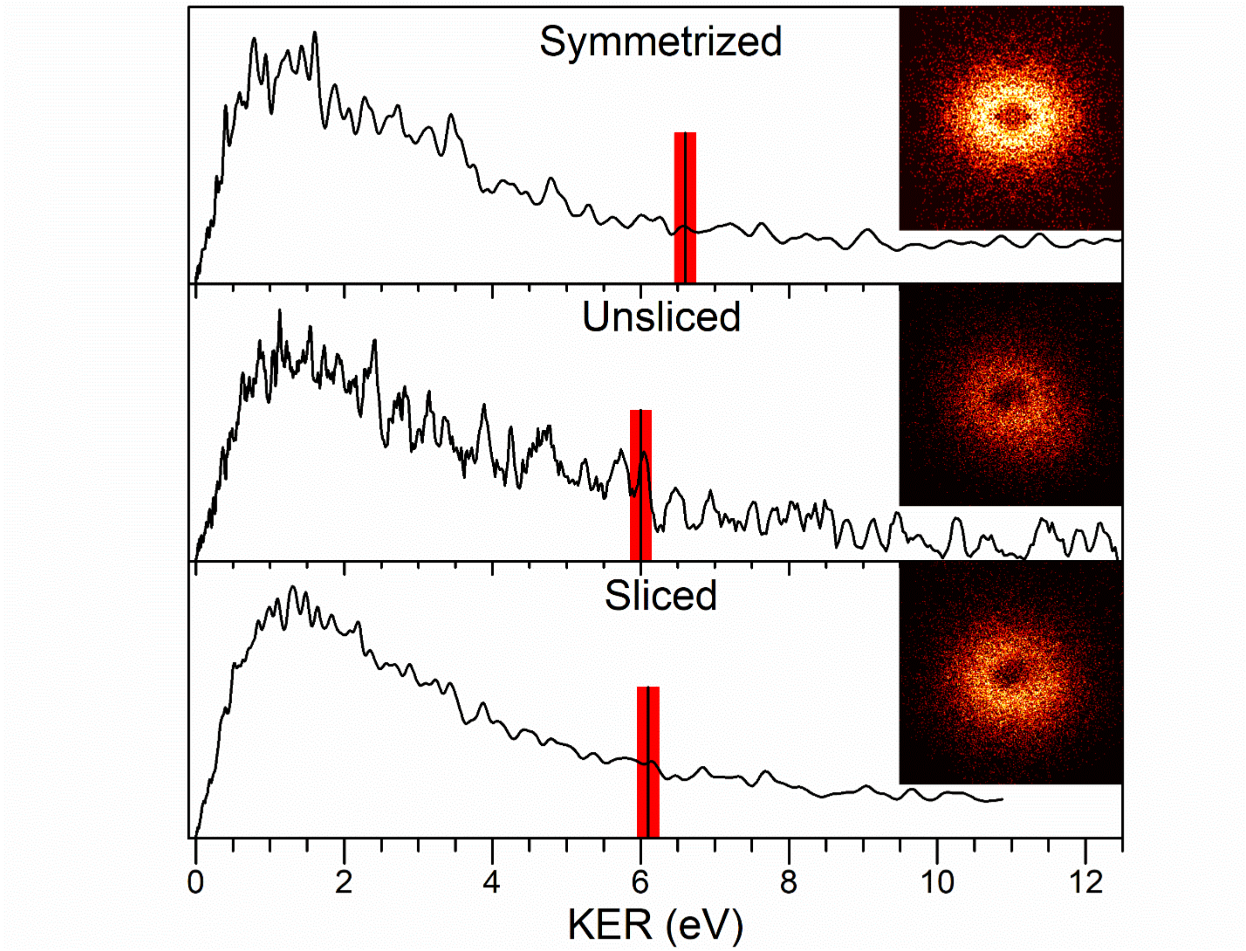

Figure S10. The total energy distribution of the sliced, unsliced, and symmetrized sliced photofragment images of Mg^{+}(benzene) → Mg + $benzene^{+}$ using 266 nm and vertical laser polarization. The black vertical line indicates the assigned value for the maximum kinetic energy, and the red box indicates the width of the instrument resolution.

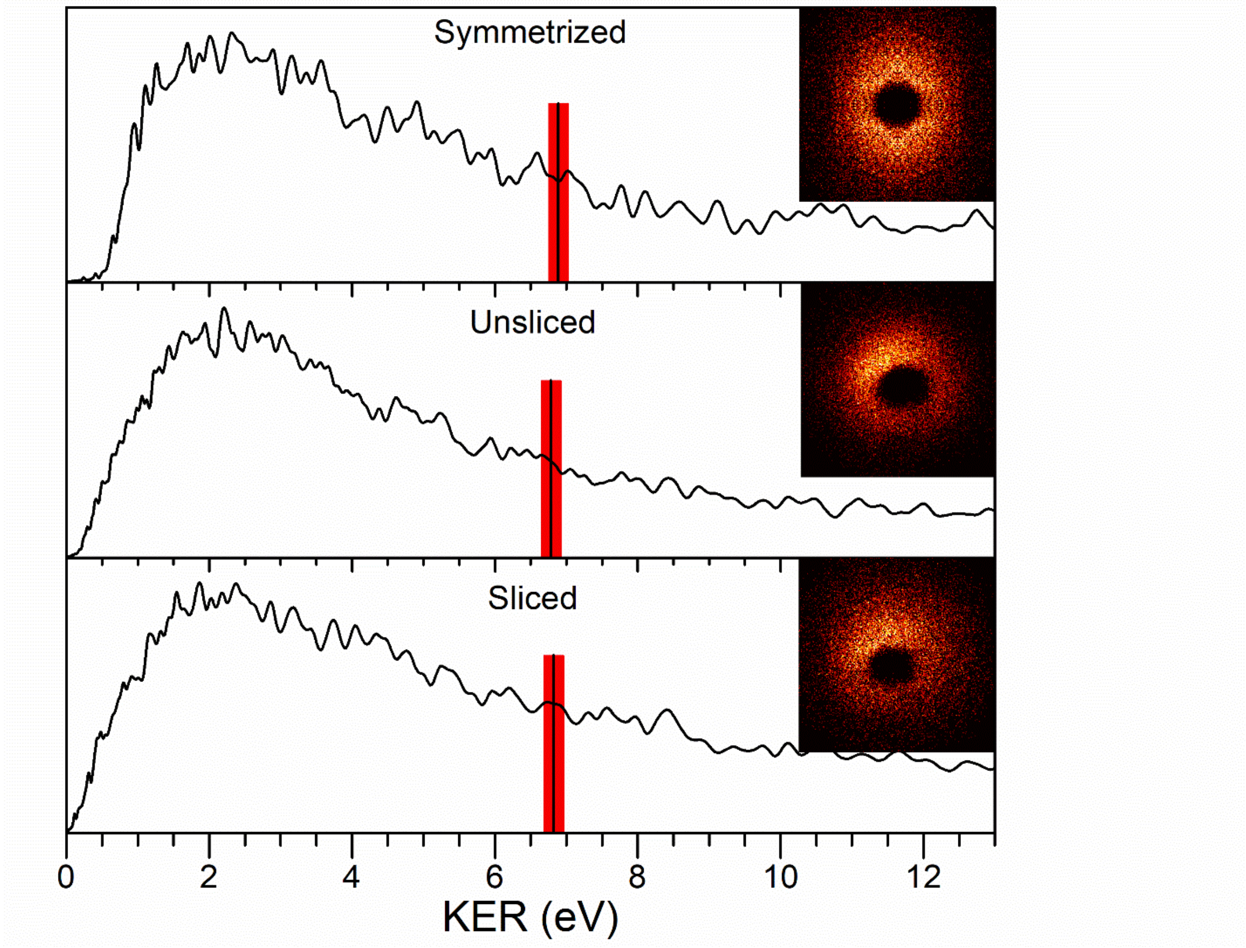

Figure S11. The total energy distribution of the sliced, unsliced, and symmetrized sliced photofragment images of Mg^{+}(benzene) → Mg + $benzene^{+}$ using 241 nm and horizontal laser polarization. The black vertical line indicates the assigned value for the maximum kinetic energy, and the red box indicates the width of the instrument resolution.

Photofragment Angular Distributions

The following equation is used for fitting angular distributions:

$$I = \frac{A}{4\pi}\left\{1 + \frac{B}{2}\left[3\cos^2\left(\frac{\theta\pi}{180} - C\right) - 1\right]\right\}$$

I is the signal intensity. The *A* parameter allows for variation of the amplitude, *B* is the *β* parameter, and *C* is a phase shift parameter correcting for rotation of the image.

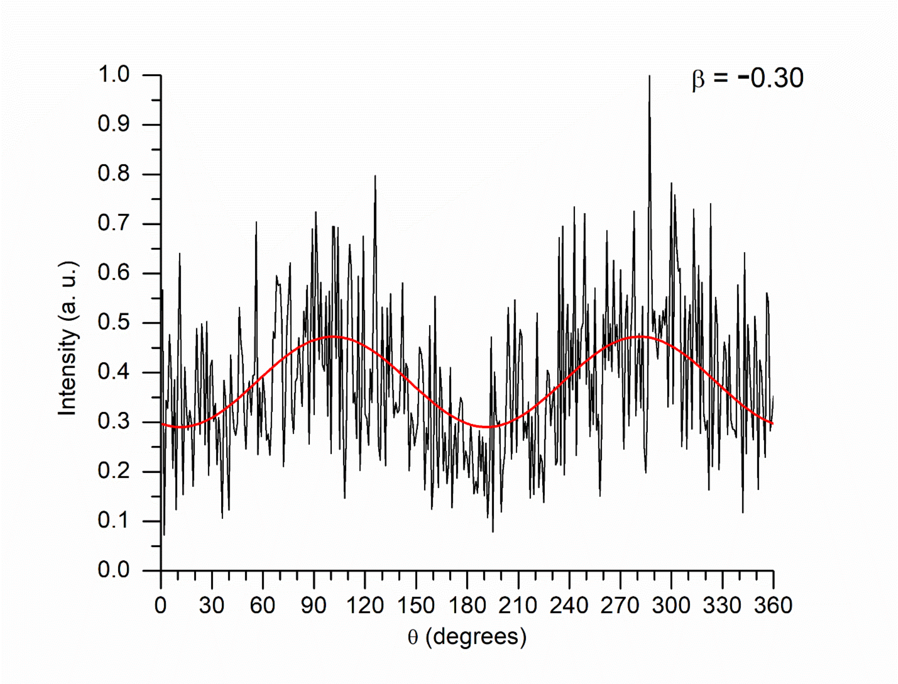

Figure S12. The angular distribution of the sliced benzene cation photofragment image from the dissociation of Mg^+(benzene) at 532 nm with vertical laser polarization. The red line is a fit with ß = –0.30.

A = 5.17352 $\pm$ 0.10270

B = $-$0.29572 $\pm$ 0.03180

C = 0.19574 $\pm$ 0.05725

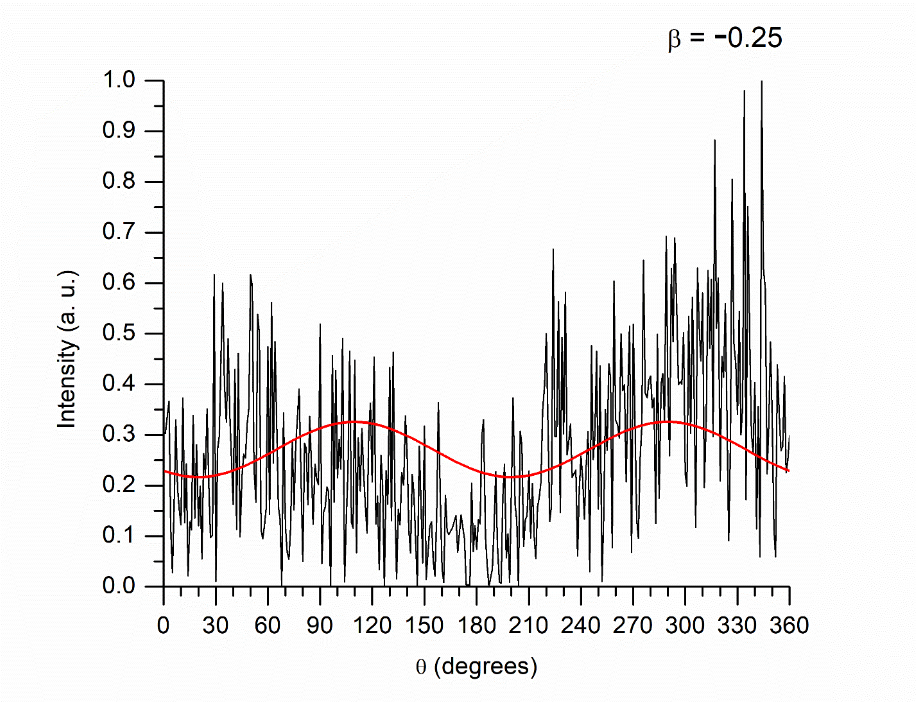

Figure S13. The angular distribution of the sliced benzene cation photofragment image from the dissociation of Mg^{+}(benzene) at 355 nm with vertical laser polarization. The red line is a fit with ß = –0.25.

A = 3.63545 $\pm$ 0.13168

B = $-$ 0.25137 $\pm$ 0.05872

C = 0.34591 $\pm$ 0.12487

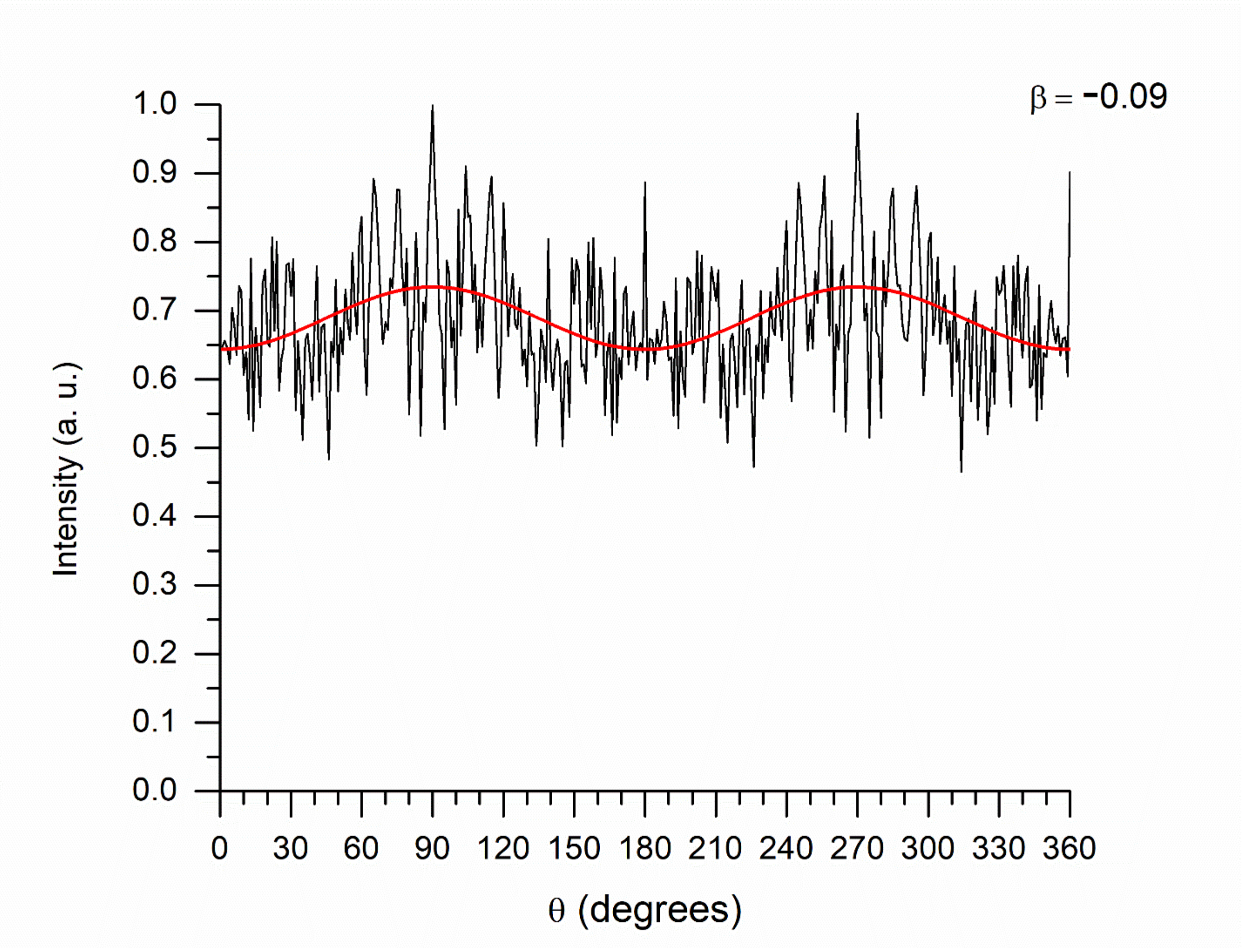

Figure S14. The angular distribution of the symmetrized benzene cation photofragment image from the dissociation of Mg^+(benzene) with horizontally polarized light at 330 nm. The red line is a fit with ß = − 0.09.

A = 8.85229 ± 0.06527

B = − 0.08620 ± 0.01232

C = − 0.00260 ± 0.07294

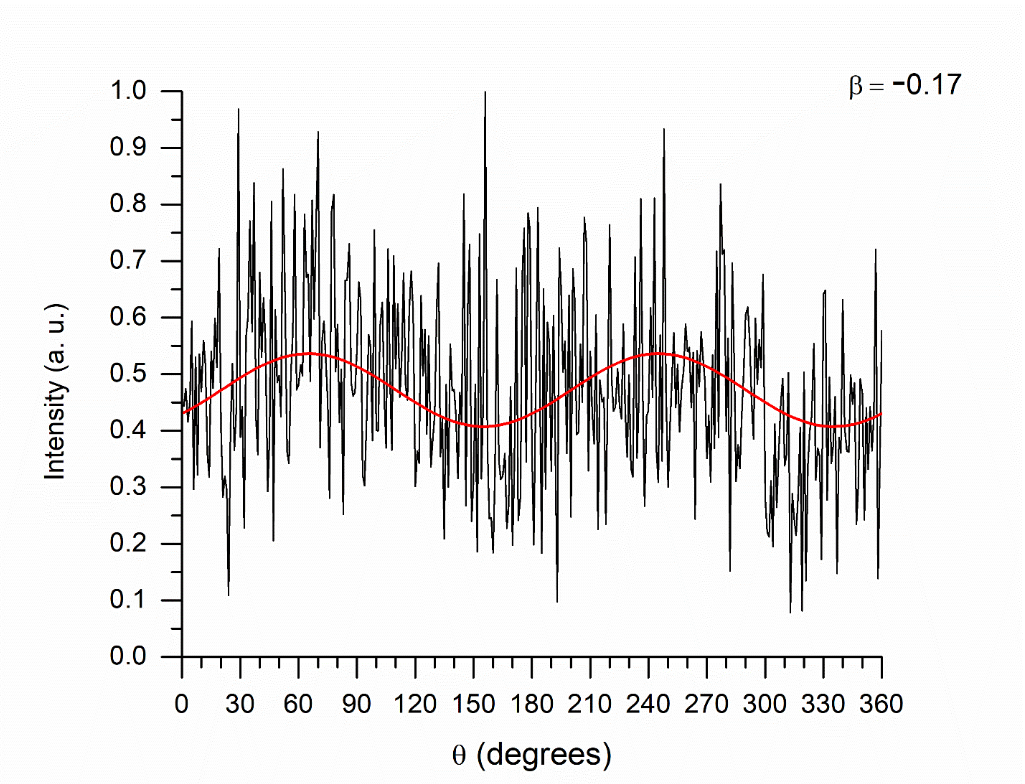

Figure S15. The angular distribution of the sliced benzene cation photofragment image from the dissociation of Mg^{+}(benzene) with horizontally polarized light at 330 nm. The red line is a fit with ß = $-$0.17.

A = 6.20212 $\pm$ 0.11890

B = $-$0.17436 $\pm$ 0.03142

C = $-$0.43375 $\pm$ 0.09376

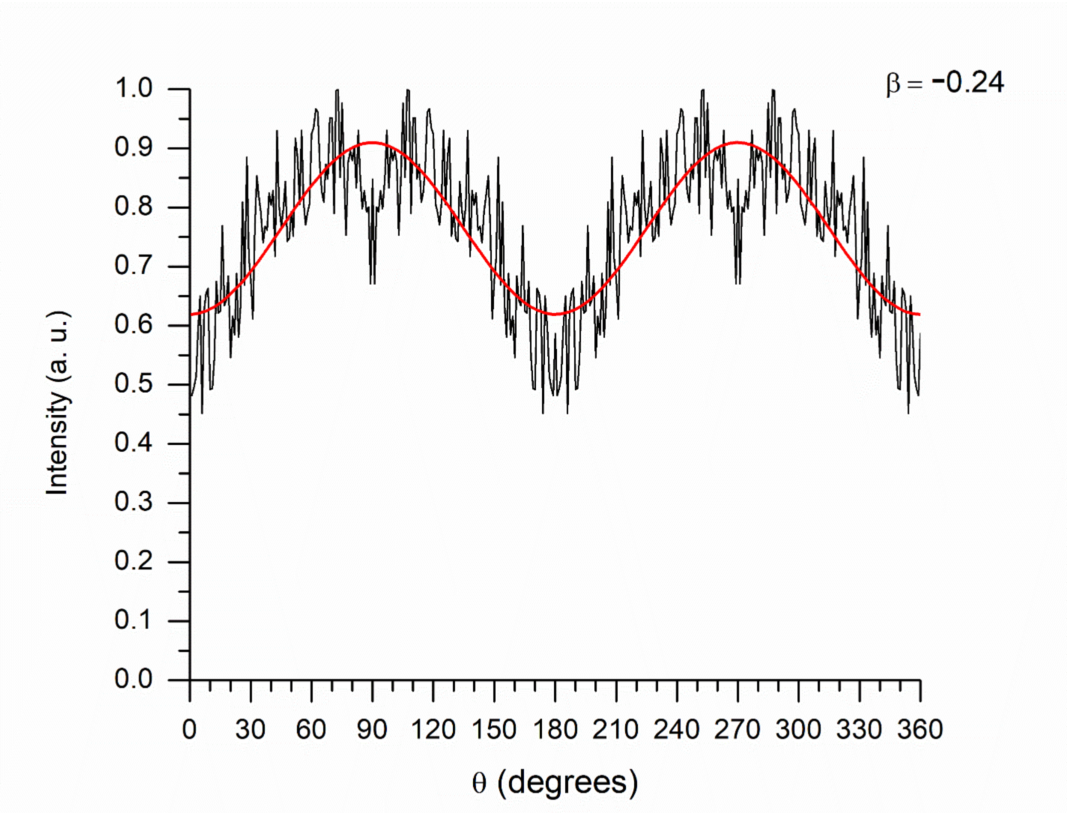

Figure S16. The angular distribution of the symmetrized benzene cation photofragment image from the dissociation of Mg^{+}(benzene) with vertically polarized light at 290 nm. The red line is a fit with ß = − 0.24.

A = 10.21537 ± 0.06125

B = − 0.23847 ± 0.00970

C = − 3.86503E-10 ± 0.02144

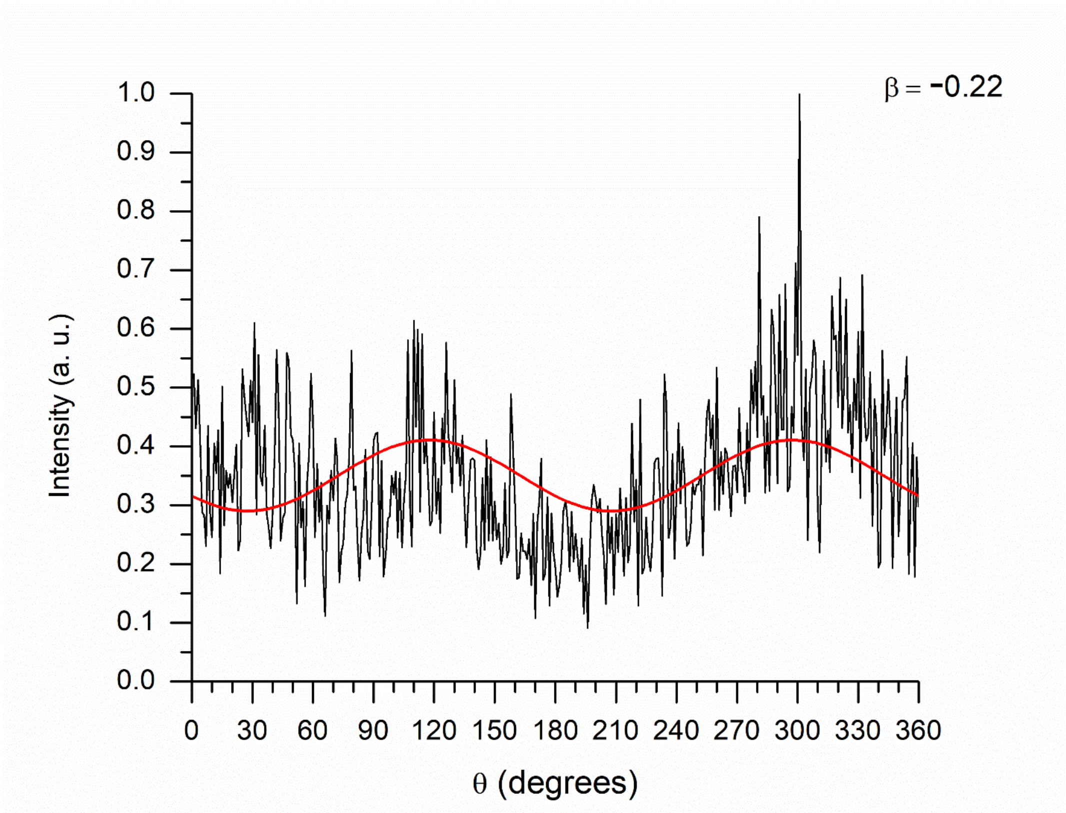

Figure S17. The angular distribution of the sliced benzene cation photofragment image from the dissociation of Mg^{+}(benzene) with vertically polarized light at 290 nm. The red line is a fit with ß = − 0.22.

A = 4.65567 ± 0.09048

B = − 0.21775 ± 0.03158

C = 0.47999 ± 0.07612

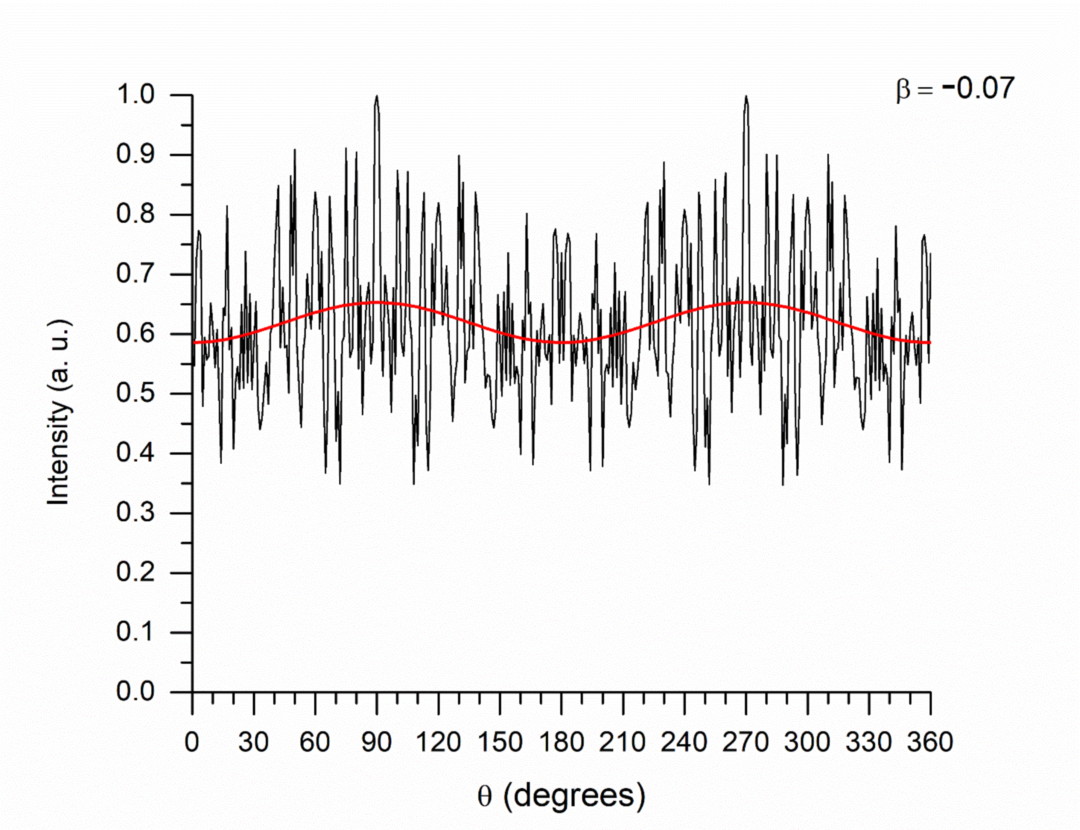

Figure S18. The angular distribution of the symmetrized benzene cation photofragment image from the dissociation of Mg^{+}(benzene) with horizontally polarized light at 290 nm. The red line is a fit with ß = − 0.07.

A = 7.92634 ± 0.09625

B = − 0.07090 ± 0.02036

C = − 0.00106 ± 0.14606

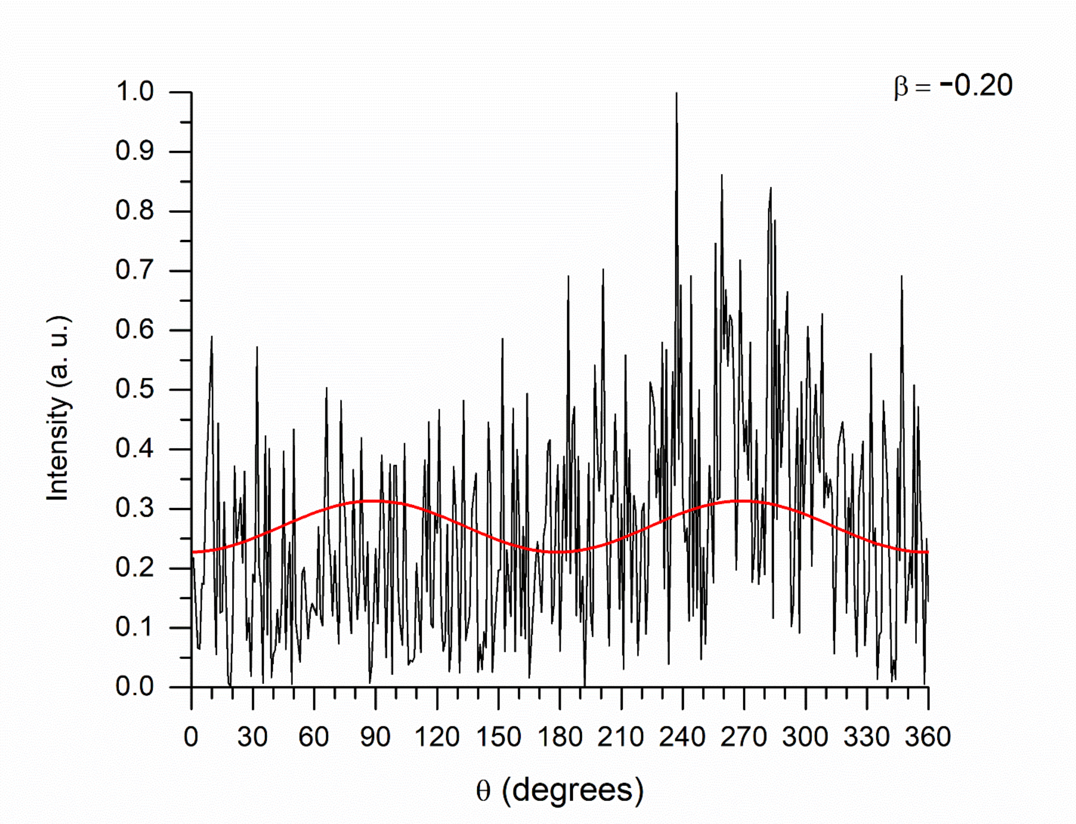

Figure S19. The angular distribution of the sliced benzene cation photofragment image from the dissociation of Mg^+(benzene) with horizontally polarized light at 290 nm. The red line is a fit with ß = −0.20.

A = 3.58067 ± 0.13900

B = −0.20180 ± 0.06334

C = −0.01975 ± 0.16430

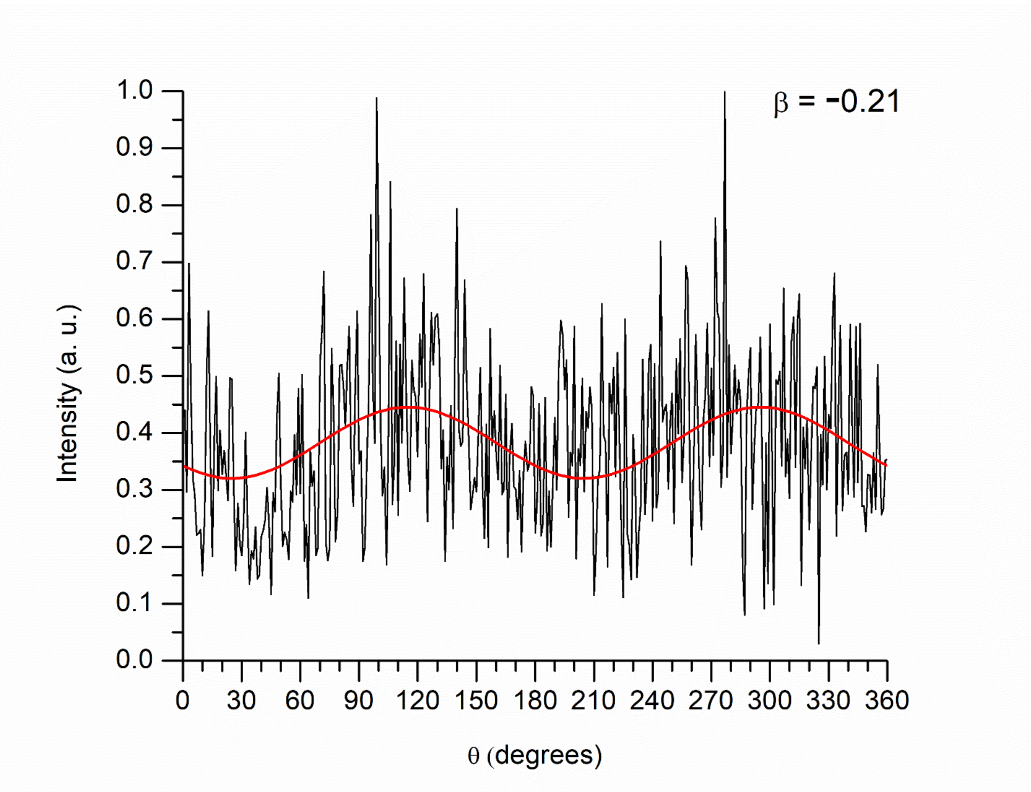

Figure S20. The angular distribution of the sliced benzene cation photofragment image from the dissociation of Mg^{+}(benzene) at 266 nm with vertical laser polarization. The red line is a fit with ß = –0.21.

A = 5.07099 $\pm$ 0.10830

B = $-$0.20671 $\pm$ 0.03477

C = 0.43647 $\pm$ 0.08811

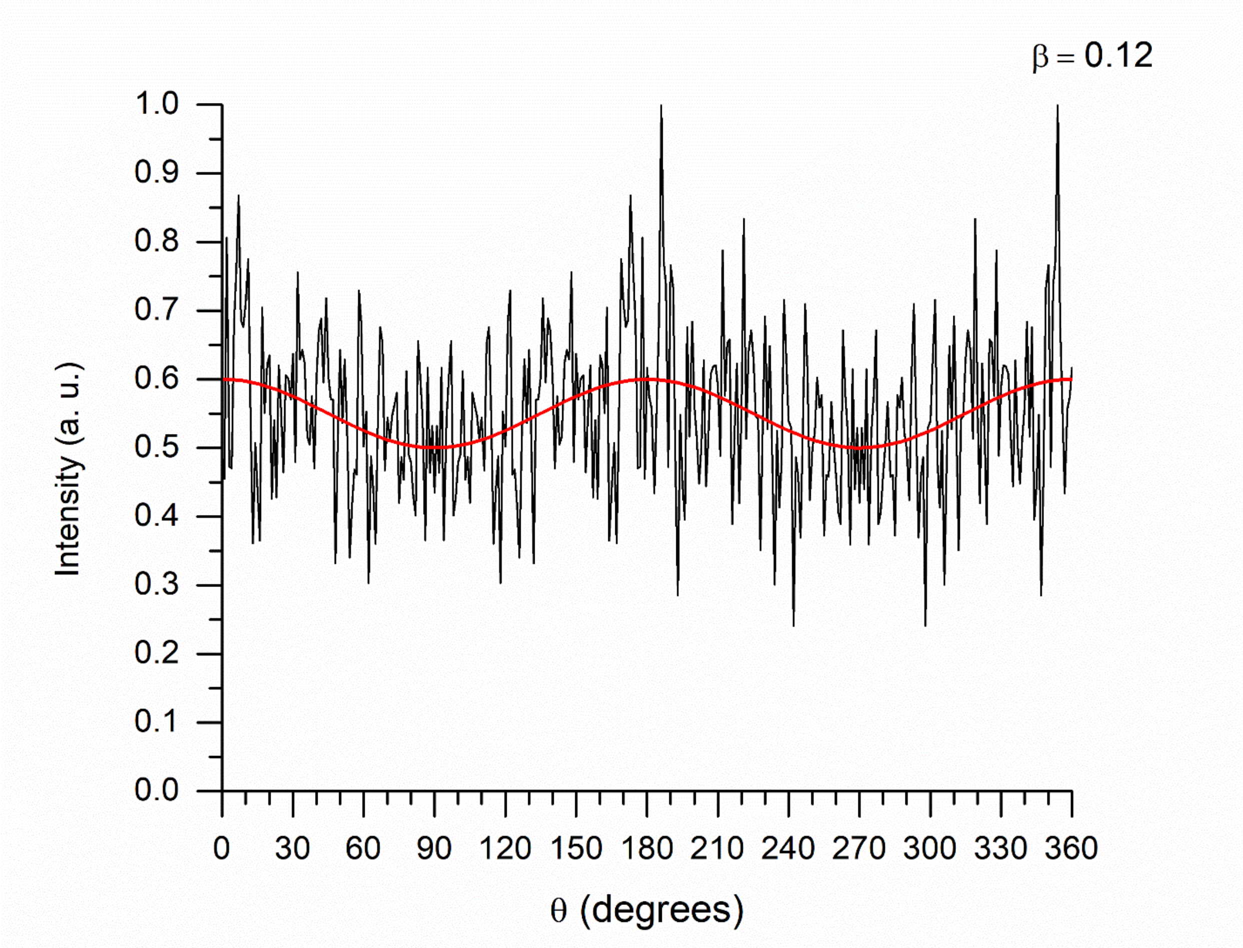

Figure S21. The angular distribution of the symmetrized benzene cation photofragment image from the dissociation of Mg^+(benzene) with horizontally polarized light at 241 nm. The red line is a fit with ß = 0.12.

A = 6.70628 $\pm$ 0.08434

B = 0.12478 $\pm$ 0.02217

C = $-$2.6415E-8 $\pm$ 0.08595

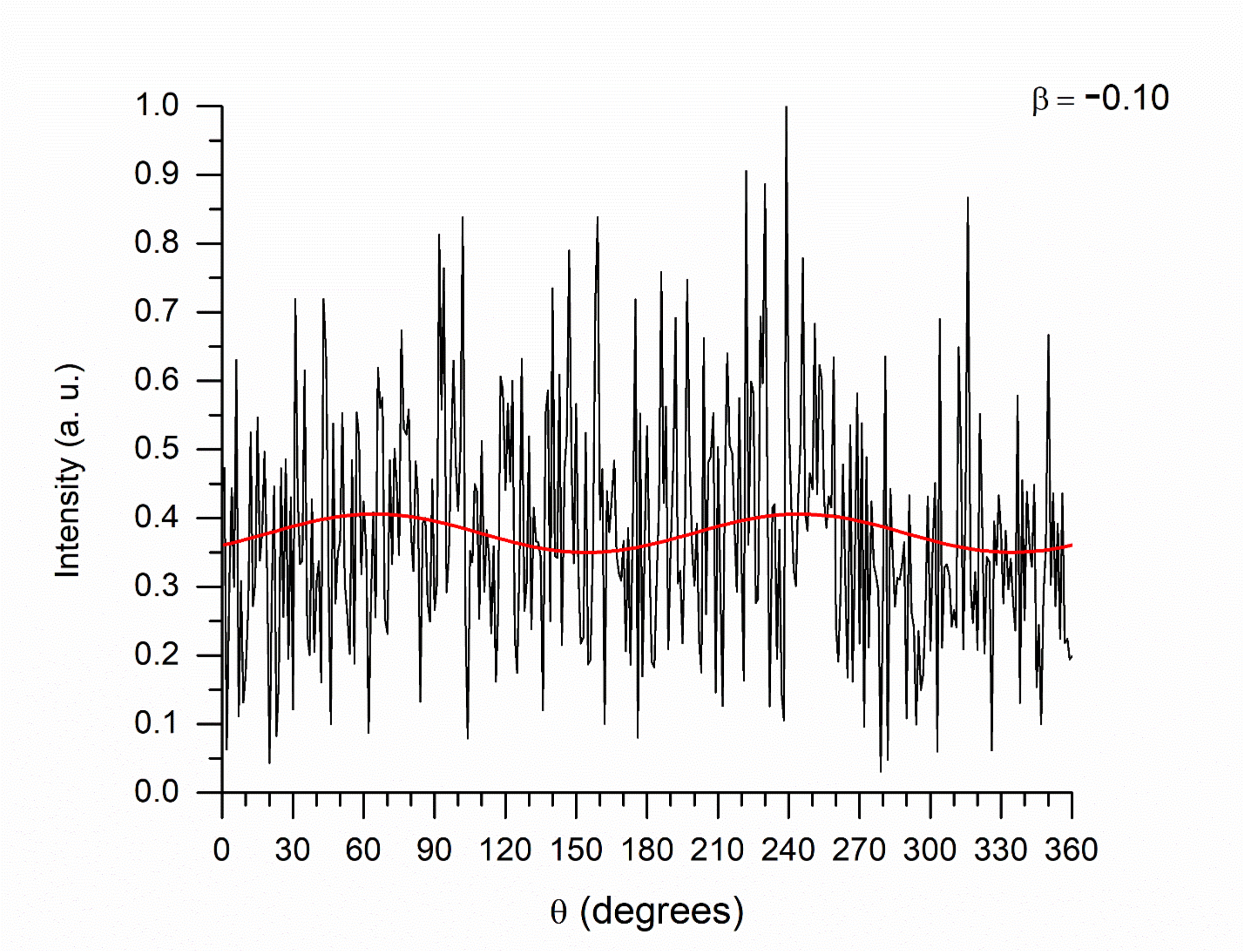

Figure S22. The angular distribution of the sliced benzene cation photofragment image from the dissociation of Mg^{+}(benzene) with horizontally polarized light at 241 nm. The red line is a fit with ß = − 0.10.

A = 4.86900 ± 0.12630

B = − 0.09614 ± 0.04324

C = − 0.44465 ± 0.23010

DFT Computations

All calculations were carried out using an "ultrafine" integration grid, and the optimization threshold for energy and structure optimizations were set to "tight." The "stable=opt" keyword was used on all structures to check for electronic wavefunction stability. All electronic energies are corrected for zero-point vibrational energy. The first 100 electronic transitions were calculated using TD-DFT. All states considered are doublets (i.e., m = 2).

Table S1. Computed energetics for Mg^+(benzene) complexes employing different DFT Functionals. Energies (zero-point corrected) are in Hartrees, except for dissociation energies which are in kcal/mol.

Energies	B3LYP	M06-L	MN15-L
benzene	-232.237193	-232.193605	-232.022182
Mg^+ ^2S	-199.806991	-199.777128	-199.729225
Mg^+(benzene)	-432.093678	-432.024948	-431.814545
Dissociation Energies			
Mg^+–(benzene)	31.06396123	34.02324068	39.62735653

Mg⁺(benzene)
B3LYP
m = 2

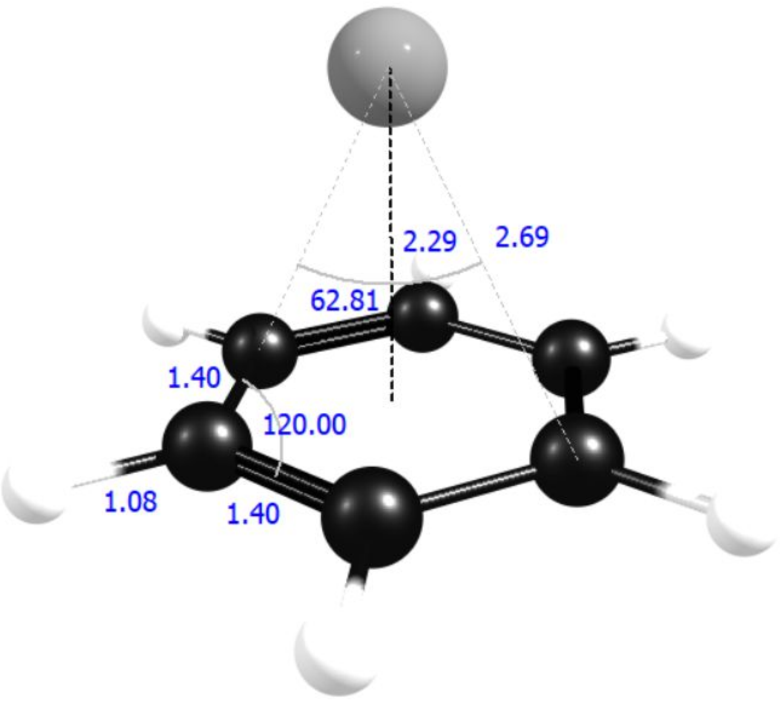

Coordinates:

6	1.308812000	0.494436000	-0.508488000
6	1.082476000	-0.886369000	-0.508483000
6	0.226146000	1.380845000	-0.508096000
1	1.919844000	-1.571956000	-0.519546000
1	0.401170000	2.448828000	-0.518835000
6	-0.226506000	-1.380769000	-0.508138000
6	-1.082833000	0.886443000	-0.507670000
1	-0.401533000	-2.448751000	-0.518910000
1	-1.920210000	1.572028000	-0.518092000
6	-1.309170000	-0.494361000	-0.507711000
1	-2.321591000	-0.876762000	-0.518155000
1	2.321226000	0.876840000	-0.519541000
12	0.000629000	-0.000131000	1.783716000

Electronic Transitions:

Wavelength, nm	Oscillator Strength
438.52	0.0000
438.51	0.0000
314.46	0.1091

314.46	0.1091
304.42	0.0000
279.47	0.0237
279.46	0.0237
251.11	0.0015
251.11	0.0015
235.93	0.0000
234.25	0.3808
221.11	0.0000
214.04	0.0331
198.62	0.0000
193.76	0.0000
193.75	0.0000
177.68	0.0240
177.07	0.0147
176.95	0.0000
176.95	0.0000
175.70	0.0000
175.70	0.0000
175.60	0.0157
175.60	0.0157
174.89	0.0000
174.08	0.0000
173.48	0.0039
171.21	0.0000
171.20	0.0000
170.82	0.6052
170.82	0.6053
170.51	0.0000
170.21	0.0000
168.19	0.0111
168.04	0.0000
168.04	0.0000
161.08	0.0000
161.08	0.0000
158.03	0.0000
157.33	0.0194
156.26	0.0000
156.26	0.0000
155.06	0.3338
150.46	0.0018
150.46	0.0018
146.62	0.0000

146.62	0.0000
146.49	0.0006
146.49	0.0006
145.63	0.0006
145.63	0.0006
143.87	0.0739
143.75	0.0068
143.75	0.0068
142.32	0.0000
139.73	0.0000
138.98	0.0020
138.98	0.0020
137.76	0.0010
137.76	0.0010
137.46	0.0000
136.95	0.0000
136.86	0.0131
136.86	0.0131
136.38	0.0004
136.38	0.0004
133.85	0.0000
132.49	0.0000
132.49	0.0000
132.47	0.0000
131.19	0.0000
130.71	0.0000
130.69	0.0064
130.69	0.0064
129.75	0.0000
128.38	0.0008
128.25	0.0000
128.25	0.0000
127.76	0.0013
127.76	0.0013
126.49	0.0000
126.41	0.0000
126.19	0.0000
126.19	0.0000
126.18	0.0011
126.18	0.0011
125.88	0.0009
125.15	0.0000
125.15	0.0000

124.22	0.0000
123.96	0.0000
123.00	0.0003
123.00	0.0003
122.94	0.0000
122.94	0.0000
122.53	0.0000
122.32	0.0089
122.32	0.0089
121.85	0.0067
121.85	0.0067

Benzene^{+}
B3LYP
m = 2

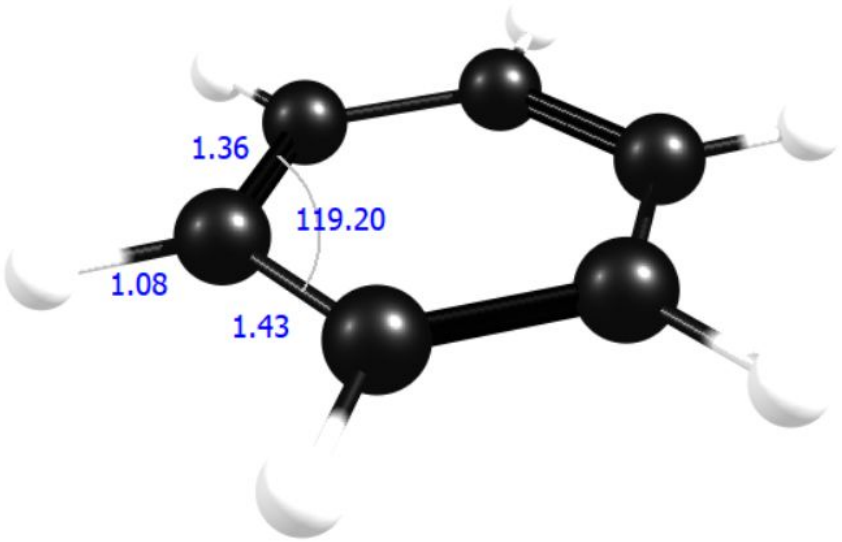

Coordinates:

6	-1.244457000	-0.682695000	0.000003000
6	0.000239000	-1.378090000	0.000008000
6	-1.244690000	0.682303000	-0.000010000
1	0.000401000	-2.461838000	-0.000006000
1	-2.168200000	1.245471000	-0.000011000
6	1.244690000	-0.682303000	0.000002000
6	-0.000239000	1.378090000	0.000002000
1	2.168200000	-1.245471000	-0.000003000
1	-0.000401000	2.461838000	0.000061000
6	1.244457000	0.682694000	-0.000010000
1	2.167781000	1.246170000	-0.000009000
1	-2.167781000	-1.246170000	-0.000005000

Electronic Transitions:

Wavelength, nm	Oscillator Strength
2120.61	0.0000
460.95	0.0000
426.23	0.0000
391.29	0.0253
271.99	0.0048
270.37	0.0180
269.69	0.0007

243.79	0.0000
223.21	0.0000
218.23	0.0514
215.20	0.0114
209.68	0.0000
185.80	0.0000
180.18	0.0012
178.38	0.3794
169.21	0.4720
169.07	0.0000
165.24	0.0000
164.89	0.0000
163.74	0.0006
160.87	0.0000
158.77	0.0000
155.29	0.0055
154.62	0.0010
150.59	0.0000
143.90	0.0000
143.32	0.0000
141.83	0.0000
141.38	0.0000
139.22	0.0000
135.43	0.0000
133.79	0.0000
131.66	0.0000
131.60	0.0000
130.01	0.0000
129.26	0.0000
127.66	0.0000
127.27	0.0008
127.16	0.0000
126.25	0.0000
125.96	0.0000
125.00	0.0000
124.77	0.0126
123.80	0.0000
123.62	0.0000
123.10	0.0000
122.03	0.0000
121.30	0.0044
121.25	0.0000
121.16	0.0000

119.48	0.0000
119.18	0.0000
118.43	0.0000
118.41	0.0000
118.39	0.0000
117.70	0.0000
117.01	0.0000
116.60	0.0000
116.46	0.0000
115.19	0.0000
114.99	0.0000
114.64	0.0000
113.67	0.0000
113.52	0.0000
112.65	0.0000
112.18	0.0000
111.62	0.0000
110.91	0.0000
109.92	0.0017
109.59	0.0000
109.42	0.0000
108.48	0.0000
107.70	0.0000
107.66	0.0000
107.49	0.0006
106.39	0.0000
105.98	0.0004
105.78	0.0003
104.58	0.0000
104.38	0.0000
102.82	0.0009
102.49	0.0009
102.40	0.0681
102.19	0.0000
101.88	0.0054
101.20	0.0001
101.19	0.0903
101.14	0.0000
101.03	0.0000
100.38	0.0000
100.36	0.1232
100.23	0.0000
100.17	0.0000

99.81	0.0000
99.70	0.0589
99.12	0.0000
98.91	0.0000
98.69	0.0000
98.22	0.0708
97.91	0.0016

Mg^+
B3LYP
m = 2

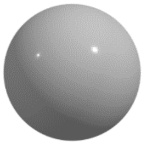

Coordinates:

12	0.000000000	0.000000000	0.000000000

Electronic Transitions:

Wavelength, nm	Oscillator Strength
261.84	0.3120
261.84	0.3120
261.84	0.3120
142.64	0.0000
142.64	0.0000
142.64	0.0000
142.64	0.0000
142.64	0.0000
138.62	0.0000
101.60	0.0003
101.60	0.0003
101.60	0.0003
64.79	0.0000
64.79	0.0000
64.79	0.0000
64.79	0.0000
64.79	0.0000
24.68	0.0658
24.68	0.0658
24.68	0.0658
10.27	0.0000
7.01	0.0000
7.01	0.0000
7.01	0.0000
7.01	0.0000
7.01	0.0000

Benzene
B3LYP
m = 1

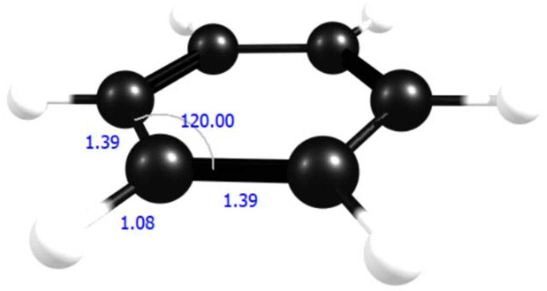

Coordinates:

6	-0.700841000	-1.201572000	0.000002000
6	0.690177000	-1.207728000	0.000001000
6	-1.391024000	0.006156000	0.000001000
1	1.227496000	-2.147985000	-0.000006000
1	-2.473971000	0.010955000	-0.000008000
6	1.391024000	-0.006156000	0.000000000
6	-0.690178000	1.207728000	0.000001000
1	2.473971000	-0.010954000	-0.000008000
1	-1.227496000	2.147985000	-0.000009000
6	0.700841000	1.201572000	0.000002000
1	1.246465000	2.137036000	-0.000006000
1	-1.246465000	-2.137036000	-0.000008000

Electronic Transitions:

Wavelength, nm	Oscillator Strength
227.84	0.0000
202.86	0.0000
174.64	0.5836
174.64	0.5836
170.35	0.0000
170.35	0.0000
162.03	0.0000
159.99	0.0000
159.99	0.0000
159.92	0.0102
152.75	0.0179

151.39	0.0000
151.39	0.0000
149.78	0.0000
138.49	0.0000
138.49	0.0000
138.41	0.0000
138.24	0.0000
138.15	0.0000
138.15	0.0000
128.43	0.0000
128.43	0.0000
127.60	0.0000
126.52	0.0000
126.52	0.0000
126.38	0.0000
126.38	0.0000
125.23	0.0000
124.27	0.0000
124.27	0.0000
121.30	0.0000
121.30	0.0000
119.30	0.0000
119.30	0.0000
119.02	0.0013
116.39	0.0000
116.28	0.0000
116.16	0.1596
116.16	0.1596
111.07	0.0000
111.07	0.0000
109.55	0.0000
109.50	0.0000
109.50	0.0000
109.11	0.0000
109.11	0.0000
107.60	0.0000
107.23	0.0003
107.23	0.0003

106.68	0.0000
106.68	0.0000
105.23	0.0000
104.58	0.0000
104.58	0.0000
104.52	0.0000
103.66	0.0705
103.66	0.0705
102.82	0.0000
102.82	0.0000
102.52	0.0000
102.52	0.0000
102.13	0.5623
102.13	0.5624
101.36	0.0000
101.36	0.0000
100.72	0.0000
99.73	0.0000
99.73	0.0000
98.84	0.0000
98.36	0.4490
98.31	0.0000
98.27	0.0000
98.27	0.0000
98.03	0.0000
98.03	0.0000
97.54	0.0000
97.00	0.0000
96.65	0.0000
96.04	0.0000
96.04	0.0000
95.95	0.0000
94.89	0.0000
94.82	0.0000
94.67	0.0156
94.53	0.0000
94.53	0.0000
92.46	0.0000

92.46	0.0000
92.28	0.5160
92.17	0.0000
91.95	0.0000
91.54	0.3051
91.54	0.3051
91.44	0.0000
89.07	0.0000
89.07	0.0000
89.00	0.0000
89.00	0.0000
88.15	0.0154
88.15	0.0154

Mg
B3LYP
m = 1

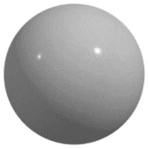

Coordinates:

12	0.000000000	0.000000000	0.000000000

Electronic Transitions:

Wavelength, nm	Oscillator Strength
269.14	0.6498
269.14	0.6498
269.14	0.6498
173.06	0.0000
173.06	0.0000
173.06	0.0000
173.06	0.0000
173.06	0.0000
172.54	0.0000
104.80	0.0006
104.80	0.0006
104.80	0.0006
62.23	0.0000
62.23	0.0000
62.23	0.0000
62.23	0.0000
62.23	0.0000
23.93	0.0472
23.93	0.0472
23.93	0.0472
10.08	0.0000
6.93	0.0000
6.93	0.0000
6.93	0.0000
6.93	0.0000
6.93	0.0000

Mg^{+}(benzene)
MN15-L
m = 2

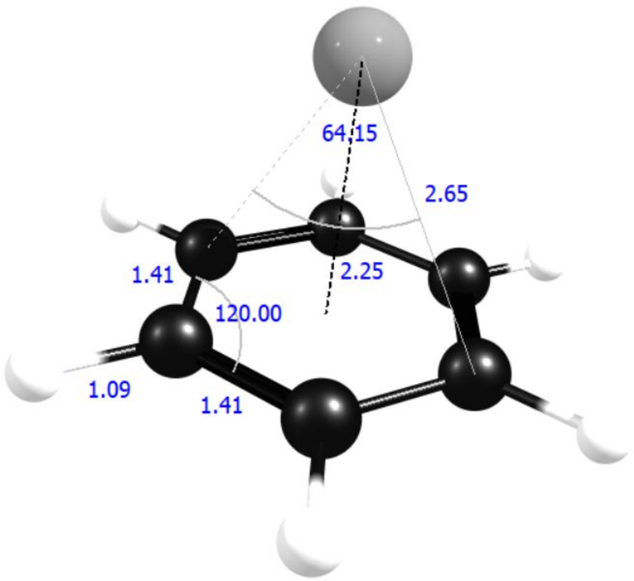

Coordinates:

6	-1.226535000	-0.694154000	-0.499325000
6	-1.214327000	0.715060000	-0.499681000
6	-0.012203000	-1.409360000	-0.499045000
1	-2.156692000	1.270329000	-0.502795000
1	-0.021850000	-2.503109000	-0.501658000
6	0.012305000	1.409064000	-0.499761000
6	1.214424000	-0.715350000	-0.499099000
1	0.021956000	2.502812000	-0.502943000
1	2.156790000	-1.270619000	-0.501757000
6	1.226639000	0.693854000	-0.499473000
1	2.178654000	1.232415000	-0.502434000
1	-2.178542000	-1.232730000	-0.502192000
12	-0.000177000	0.000519000	1.749341000

Electronic Transitions:

Wavelength, nm	Oscillator Strength
553.35	0.0000
553.24	0.0000
325.80	0.1307
325.79	0.1307

281.10	0.0000
245.03	0.2257
240.84	0.0014
240.83	0.0014
234.15	0.2083
230.34	0.0144
230.32	0.0144
221.73	0.0000
210.92	0.0000
194.81	0.0000
186.21	0.0189
185.96	0.0002
185.96	0.0002
181.48	0.0000
178.29	0.0000
178.29	0.0000
178.18	0.0676
177.61	0.0000
177.61	0.0000
176.74	0.0000
176.73	0.0000
175.34	0.0000
172.68	0.0000
171.55	0.0000
171.55	0.0000
170.93	0.0000
170.14	0.0000
167.04	0.0000
167.04	0.0000
166.59	0.0100
164.55	0.6567
164.55	0.6567
157.16	0.0000
157.16	0.0000
152.47	0.0000
152.47	0.0000
152.01	0.0000
151.76	0.1160

151.49	0.0000
149.26	0.0000
149.25	0.0000
148.20	0.0181
146.13	0.0003
146.12	0.0003
144.13	0.0000
144.07	0.0006
144.07	0.0006
143.84	0.0000
143.05	0.2279
143.02	0.0027
143.01	0.0027
139.95	0.0047
139.94	0.0047
137.99	0.0021
137.99	0.0021
134.60	0.0015
134.60	0.0015
132.95	0.0000
131.44	0.0000
131.44	0.0000
130.92	0.0000
130.91	0.0000
130.60	0.0000
129.38	0.0000
128.88	0.0000
128.65	0.0041
128.65	0.0041
127.63	0.0000
127.49	0.0039
127.49	0.0039
127.08	0.0000
125.90	0.0000
125.90	0.0000
124.84	0.0000
124.83	0.0000
123.27	0.0000

122.79	0.0000
122.79	0.0000
122.57	0.0000
122.56	0.0000
122.50	0.0113
122.50	0.0113
122.18	0.0003
121.57	0.0037
121.57	0.0037
121.03	0.0000
121.03	0.0000
120.99	0.0000
120.92	0.0000
120.90	0.0012
120.90	0.0013
120.80	0.0000
120.79	0.0000
120.05	0.0027
120.05	0.0025
120.04	0.0000

Benzene$^+$
MN15-L
m = 2

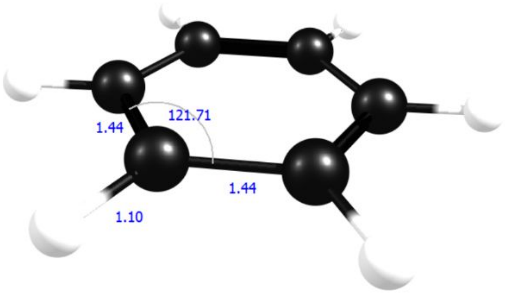

Coordinates:

6	-1.254941000	-0.687034000	0.000010000
6	-0.000128000	-1.386912000	-0.000005000
6	-1.254821000	0.687242000	-0.000012000
1	-0.000216000	-2.482242000	-0.000029000
1	-2.186721000	1.258465000	-0.000034000
6	1.254820000	-0.687242000	0.000009000
6	0.000128000	1.386912000	0.000011000
1	2.186720000	-1.258465000	0.000026000
1	0.000216000	2.482243000	0.000049000
6	1.254941000	0.687034000	-0.000013000
1	2.186941000	1.258094000	-0.000031000
1	-2.186941000	-1.258093000	0.000023000

Electronic Transitions:

Wavelength, nm	Oscillator Strength
1353.94	0.0000
434.44	0.0000
398.47	0.0000
393.31	0.0277
259.04	0.0060
255.27	0.0007
248.85	0.0303
231.50	0.0000
210.79	0.0368
208.59	0.0000
201.39	0.0000

200.05	0.0015
180.97	0.0000
175.45	0.0013
167.95	0.4047
163.42	0.0000
162.72	0.5253
158.55	0.0004
157.88	0.0000
157.18	0.0000
154.65	0.0000
152.98	0.0046
152.37	0.0000
149.39	0.0000
146.85	0.0029
140.82	0.0000
138.52	0.0000
138.32	0.0000
138.03	0.0000
135.28	0.0000
132.64	0.0000
131.09	0.0000
127.58	0.0000
124.43	0.0000
124.06	0.0000
123.51	0.0000
123.31	0.0000
122.48	0.0020
122.47	0.0000
122.26	0.0000
121.48	0.0000
121.15	0.0000
120.60	0.0000
120.46	0.0071
119.86	0.0000
118.82	0.0000
117.91	0.0000
117.41	0.0000
117.15	0.0050

116.66	0.0000
116.11	0.0000
115.74	0.0000
115.66	0.0000
114.90	0.0000
114.46	0.0000
113.69	0.0000
113.42	0.0000
113.10	0.0000
112.98	0.0000
111.83	0.0000
111.62	0.0000
111.06	0.0000
110.80	0.0000
110.60	0.0000
110.58	0.0000
109.99	0.0000
109.57	0.0020
108.89	0.0000
108.14	0.0000
108.05	0.0000
106.40	0.0000
106.30	0.0000
106.26	0.0000
105.47	0.0023
104.76	0.0000
104.41	0.0000
104.08	0.0033
103.57	0.0023
103.39	0.0000
103.38	0.0000
102.16	0.0025
101.92	0.0865
101.34	0.0532
100.72	0.0063
100.36	0.1441
99.82	0.0004
99.81	0.0000

99.46	0.0000
99.37	0.0000
99.25	0.1009
98.42	0.0000
98.12	0.0000
98.11	0.0000
97.40	0.0000
97.36	0.0000
97.24	0.0200
96.63	0.0000
96.54	0.0000
96.30	0.0000
95.83	0.0000

Mg^+
MN15-L
m = 2

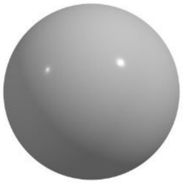

Coordinates:

12	0.000000000	0.000000000	0.000000000

Electronic Transitions:

Wavelength, nm	Oscillator Strength
267.19	0.3294
267.19	0.3294
267.19	0.3294
146.01	0.0000
145.97	0.0000
145.97	0.0000
145.97	0.0000
145.97	0.0000
145.97	0.0000
102.11	0.0052
102.11	0.0052
102.11	0.0052
63.63	0.0000
63.63	0.0000
63.63	0.0000
63.63	0.0000
63.63	0.0000
23.87	0.0408
23.87	0.0408
23.87	0.0408
9.81	0.0000
6.78	0.0000
6.78	0.0000
6.78	0.0000
6.78	0.0000
6.78	0.0000

Benzene
MN15-L
m = 1

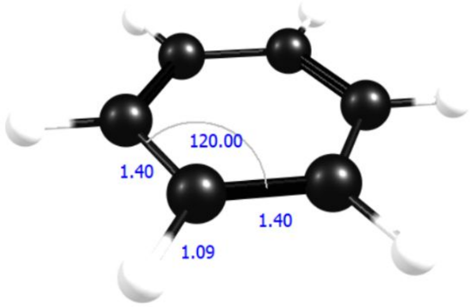

Coordinates:

6	-0.764179000	-1.173188000	0.000000000
6	0.633939000	-1.248378000	-0.000003000
6	-1.398153000	0.075191000	-0.000002000
1	1.129461000	-2.224287000	0.000003000
1	-2.491079000	0.133991000	-0.000009000
6	1.398153000	-0.075191000	0.000003000
6	-0.633939000	1.248378000	0.000003000
1	2.491079000	-0.133991000	-0.000003000
1	-1.129461000	2.224287000	0.000004000
6	0.764179000	1.173188000	-0.000002000
1	1.361526000	2.090314000	-0.000005000
1	-1.361526000	-2.090314000	0.000007000

Electronic Transitions:

Wavelength, nm	Oscillator Strength
216.59	0.0000
198.76	0.0000
166.70	0.6210
166.70	0.6211
165.85	0.0000
165.84	0.0000
160.31	0.0000
156.88	0.0000
156.88	0.0000
156.35	0.0091

145.94	0.0182
145.62	0.0000
145.62	0.0000
144.51	0.0000
135.20	0.0000
135.20	0.0000
133.76	0.0000
133.76	0.0000
133.73	0.0000
133.10	0.0000
127.58	0.0000
127.57	0.0000
126.42	0.0000
126.41	0.0000
124.74	0.0000
123.11	0.0000
123.01	0.0000
123.01	0.0000
119.02	0.0000
119.01	0.0000
117.61	0.0032
116.53	0.0000
116.52	0.0000
116.42	0.0000
115.91	0.0000
115.90	0.0000
115.66	0.1816
115.66	0.1815
115.32	0.0000
108.77	0.0000
108.75	0.0000
108.75	0.0000
108.24	0.0000
108.24	0.0000
107.35	0.0000
107.01	0.0000
107.01	0.0000
104.61	0.2374

104.61	0.2373
103.75	0.1262
103.75	0.1262
101.62	0.3636
101.62	0.3637
101.47	0.0000
101.47	0.0000
101.19	0.0000
100.75	0.0000
100.75	0.0000
100.61	0.0000
100.61	0.0000
100.60	0.0000
99.87	0.0000
99.87	0.0000
98.48	0.0000
97.60	0.0000
97.60	0.0000
97.05	0.0000
97.05	0.0000
96.83	0.0000
96.42	0.0000
96.40	0.0000
96.40	0.0000
95.96	0.0000
95.87	0.0000
95.86	0.0000
95.28	0.4875
95.27	0.0000
94.99	0.0000
93.56	0.0000
93.56	0.0000
92.72	0.0000
92.01	0.0000
91.90	0.0793
91.58	0.0000
91.36	0.0000
91.36	0.0000

91.28	0.0000
91.18	0.0000
91.18	0.0000
90.79	0.3595
90.79	0.3637
90.78	0.0043
90.43	0.0000
90.23	0.5317
87.95	0.0000
87.95	0.0000
87.61	0.0000
87.61	0.0000
86.34	0.1038
86.34	0.1039

Mg
MN15-L
m = 1

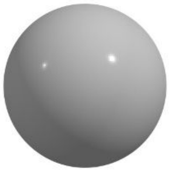

Coordinates:

12	0.000000000	0.000000000	0.000000000

Electronic Transitions:

Wavelength, nm	Oscillator Strength
272.79	0.6946
272.79	0.6946
272.79	0.6946
173.53	0.0000
173.53	0.0000
173.53	0.0000
173.53	0.0000
173.53	0.0000
170.07	0.0000
99.30	0.0044
99.30	0.0044
99.30	0.0044
58.99	0.0000
58.99	0.0000
58.99	0.0000
58.99	0.0000
58.99	0.0000
23.33	0.0246
23.33	0.0246
23.33	0.0246
9.66	0.0000
6.71	0.0000
6.71	0.0000
6.71	0.0000
6.71	0.0000
6.71	0.0000

Mg⁺(benzene)
M06-L
m = 2

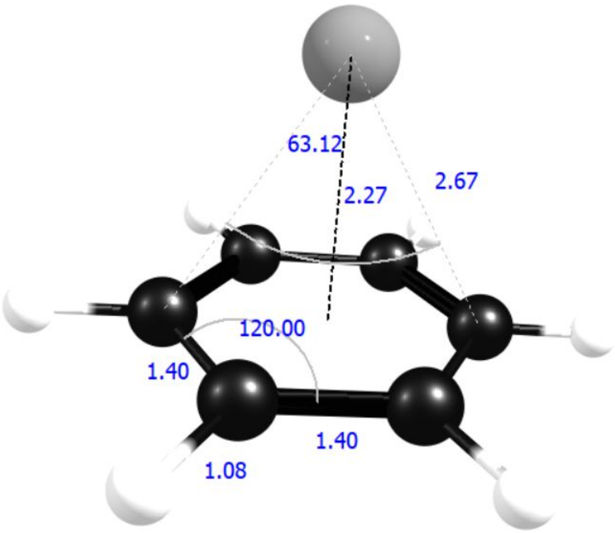

Coordinates:

6	0.197688000	-1.380495000	-0.506410000
6	-1.100112000	-0.867278000	-0.490654000
6	1.291097000	-0.513157000	-0.519468000
1	-1.947930000	-1.539749000	-0.491251000
1	2.297144000	-0.911092000	-0.542318000
6	-1.304563000	0.513381000	-0.487907000
6	1.086642000	0.867521000	-0.516692000
1	-2.310884000	0.911288000	-0.486382000
1	1.934182000	1.540017000	-0.537387000
6	-0.211194000	1.380723000	-0.500910000
1	-0.369791000	2.451136000	-0.509423000
1	0.356083000	-2.450897000	-0.519185000
12	0.023654000	-0.000407000	1.768183000

Electronic Transitions:

Wavelength, nm	Oscillator Strength
481.23	0.0000
481.17	0.0000
308.52	0.1407
307.90	0.1402
299.66	0.0000

264.57	0.0074
264.16	0.0072
247.12	0.0029
247.01	0.0030
233.82	0.0000
228.31	0.3397
220.06	0.0000
210.14	0.0098
197.90	0.0000
197.28	0.0000
197.26	0.0000
181.22	0.0000
181.14	0.0000
180.71	0.0000
180.71	0.0000
179.72	0.0000
177.32	0.0354
176.77	0.0000
173.75	0.0001
173.74	0.0000
172.79	0.0026
172.61	0.0002
172.60	0.0000
172.01	0.0124
171.99	0.0124
170.56	0.2052
169.72	0.0001
167.42	0.6009
167.41	0.6012
166.41	0.0003
166.32	0.0001
165.95	0.0000
163.93	0.0149
163.19	0.0001
163.11	0.0001
158.22	0.2411
156.81	0.0000
156.79	0.0008

148.36	0.0001
148.36	0.0001
147.74	0.0000
147.67	0.0000
145.55	0.0012
145.55	0.0012
143.14	0.0000
142.73	0.0000
142.26	0.0013
142.25	0.0013
142.11	0.0240
142.08	0.0000
141.31	0.0001
141.31	0.0001
141.13	0.0000
141.04	0.0043
141.00	0.0043
139.04	0.0029
139.04	0.0029
138.81	0.0000
138.81	0.0000
138.74	0.0000
138.56	0.0000
137.77	0.0000
137.77	0.0000
135.96	0.0000
135.96	0.0000
135.83	0.0000
135.32	0.0000
134.98	0.0000
133.96	0.0000
133.95	0.0051
133.95	0.0051
133.34	0.0000
130.63	0.0000
130.63	0.0000
128.57	0.0000
128.57	0.0000

126.04	0.0001
126.03	0.0001
123.97	0.0000
123.96	0.0000
123.89	0.0010
123.88	0.0010
122.41	0.0000
121.83	0.0000
121.83	0.0000
121.58	0.0000
121.58	0.0000
120.77	0.0001
120.76	0.0000
120.62	0.0040
120.62	0.0000
120.56	0.0000
120.54	0.0024
120.40	0.0035
120.25	0.0001

Benzene^{+}
M06-L
m = 2

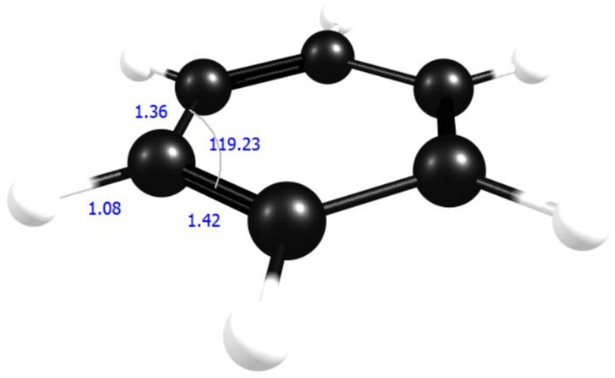

Coordinates:

6	1.240593000	0.681134000	-0.000008000
6	0.000033000	1.375403000	0.000001000
6	1.240564000	-0.681183000	0.000001000
1	0.000062000	2.459805000	0.000037000
1	2.164208000	-1.243700000	0.000011000
6	-1.240564000	0.681183000	-0.000004000
6	-0.000033000	-1.375403000	0.000004000
1	-2.164208000	1.243700000	-0.000017000
1	-0.000062000	-2.459806000	-0.000021000
6	-1.240593000	-0.681134000	0.000005000
1	-2.164284000	-1.243574000	-0.000002000
1	2.164284000	1.243574000	-0.000005000

Electronic Transitions:

Wavelength, nm	Oscillator Strength
1575.06	0.0000
477.74	0.0000
446.95	0.0000
411.85	0.0224
277.04	0.0006
269.09	0.0055
265.26	0.0207
254.96	0.0000
221.77	0.0000

219.93	0.0000
218.63	0.0418
211.35	0.0078
193.12	0.0000
185.73	0.0011
173.97	0.3731
173.21	0.0000
170.16	0.0000
168.17	0.0005
165.86	0.4768
163.93	0.0000
163.91	0.0000
163.00	0.0000
159.19	0.0044
158.10	0.0026
156.69	0.0000
146.67	0.0000
144.70	0.0000
143.88	0.0000
140.51	0.0000
137.58	0.0000
137.03	0.0000
134.23	0.0000
133.99	0.0000
131.91	0.0000
131.13	0.0000
130.49	0.0000
130.08	0.0000
129.37	0.0000
128.05	0.0000
127.48	0.0000
127.16	0.0000
124.99	0.0000
124.83	0.0000
123.25	0.0000
122.96	0.0000
122.62	0.0007
120.84	0.0000

120.15	0.0039
119.41	0.0000
119.13	0.0000
117.75	0.0000
116.94	0.0000
116.92	0.0000
116.69	0.0000
116.14	0.0021
115.99	0.0000
115.20	0.0000
114.60	0.0000
114.34	0.0000
114.17	0.0000
113.91	0.0000
113.32	0.0000
113.03	0.0000
111.43	0.0000
111.34	0.0000
111.23	0.0000
110.44	0.0000
110.33	0.0000
110.02	0.0006
109.26	0.0000
108.88	0.0000
107.92	0.0000
106.61	0.0000
106.55	0.0001
106.55	0.0000
105.83	0.0000
105.03	0.0000
104.80	0.0018
104.57	0.0044
104.19	0.0015
103.97	0.0000
103.76	0.0000
102.98	0.0504
102.57	0.0004
102.23	0.0059

102.12	0.0000
101.75	0.0864
101.55	0.0000
101.15	0.0586
100.34	0.0816
100.33	0.0000
99.85	0.0000
99.33	0.0000
99.07	0.0000
98.94	0.0124
98.93	0.0000
98.15	0.0000
98.07	0.0000
97.81	0.0000
97.74	0.0000

Mg^+
M06-L
m = 2

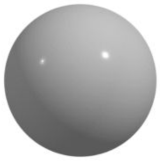

Coordinates:
12 0.000000000 0.000000000 0.000000000

Electronic Transitions:

Wavelength, nm	Oscillator Strength
270.28	0.3219
270.28	0.3219
270.28	0.3219
147.68	0.0000
147.68	0.0000
147.68	0.0000
147.68	0.0000
147.68	0.0000
140.41	0.0000
102.98	0.0002
102.98	0.0002
102.98	0.0002
64.19	0.0000
64.19	0.0000
64.19	0.0000
64.19	0.0000
64.19	0.0000
24.48	0.0673
24.48	0.0673
24.48	0.0673
10.15	0.0000
6.96	0.0000
6.96	0.0000
6.96	0.0000
6.96	0.0000
6.96	0.0000

Benzene
M06-L
m = 1

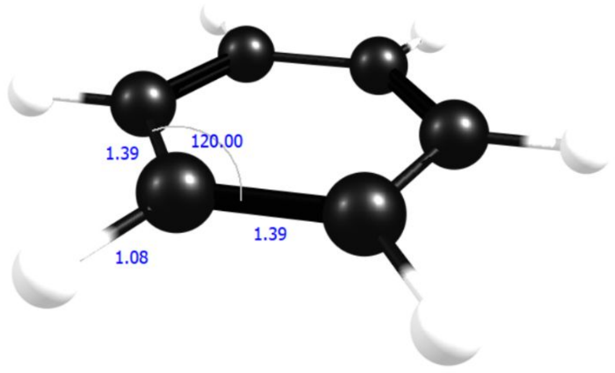

Coordinates:

6	0.159897000	1.377944000	0.000000000
6	1.273346000	0.550507000	-0.000002000
6	-1.113429000	0.827459000	0.000000000
1	2.267098000	0.980190000	0.000004000
1	-1.982376000	1.473292000	-0.000005000
6	1.113429000	-0.827458000	-0.000001000
6	-1.273345000	-0.550508000	0.000003000
1	1.982375000	-1.473294000	0.000006000
1	-2.267100000	-0.980187000	-0.000003000
6	-0.159898000	-1.377944000	-0.000001000
1	-0.284681000	-2.453397000	-0.000003000
1	0.284683000	2.453396000	0.000003000

Electronic Transitions:

Wavelength, nm	Oscillator Strength
226.34	0.0000
201.43	0.0000
170.58	0.5737
170.58	0.5738
166.04	0.0000
162.73	0.0000
162.73	0.0000
162.36	0.0092
161.41	0.0000

161.40	0.0000
142.62	0.0000
142.62	0.0000
142.39	0.0138
142.18	0.0000
141.19	0.0000
141.19	0.0000
130.88	0.0000
130.74	0.0000
130.74	0.0000
130.00	0.0000
128.93	0.0000
128.75	0.0000
128.06	0.0000
128.06	0.0000
126.12	0.0000
126.12	0.0000
124.75	0.0000
124.74	0.0000
120.35	0.0000
120.35	0.0000
120.06	0.0000
120.05	0.0000
118.63	0.0000
118.63	0.0000
117.28	0.0013
114.93	0.0000
114.72	0.1196
114.72	0.1196
114.48	0.0000
113.27	0.0000
113.27	0.0000
107.94	0.0000
107.15	0.0000
107.15	0.0000
106.99	0.0000
106.99	0.0000
106.01	0.0000

105.87	0.0000
105.86	0.0000
104.48	0.1117
104.47	0.1113
103.04	0.1672
103.04	0.1671
101.21	0.3688
101.21	0.3691
100.97	0.0000
100.93	0.0000
100.93	0.0000
100.12	0.0000
100.12	0.0000
100.03	0.0000
100.03	0.0000
99.72	0.0000
99.33	0.0000
99.33	0.0000
98.55	0.0000
97.91	0.0000
97.91	0.0000
97.34	0.0000
97.31	0.9127
97.00	0.0000
96.69	0.0000
96.62	0.0000
96.36	0.0000
96.36	0.0000
95.86	0.0000
95.85	0.0000
95.68	0.0000
94.22	0.0000
93.35	0.0000
93.35	0.0000
92.92	0.0000
92.83	0.0202
92.71	0.0000
92.71	0.0000

91.51	0.0000
91.51	0.0000
91.38	0.0000
91.34	0.1465
90.85	0.0000
90.65	0.0000
90.52	0.3552
90.52	0.3553
90.09	0.0000
90.09	0.0000
89.67	0.0000
88.88	0.0000
88.88	0.0000
88.00	0.0000
88.00	0.0000

Mg
M06-L
m = 1

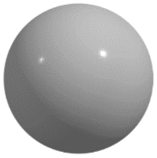

Coordinates:

12	0.000000000	0.000000000	0.000000000

Electronic Transitions:

Wavelength, nm	Oscillator Strength
275.14	0.6860
275.14	0.6860
275.14	0.6860
181.82	0.0000
181.82	0.0000
181.82	0.0000
181.82	0.0000
181.82	0.0000
171.54	0.0000
102.91	0.0065
102.91	0.0065
102.91	0.0065
60.01	0.0000
60.01	0.0000
60.01	0.0000
60.01	0.0000
60.01	0.0000
24.30	0.0583
24.30	0.0583
24.30	0.0584
10.01	0.0000
6.87	0.0000
6.87	0.0000
6.87	0.0000
6.87	0.0000
6.87	0.0000
